# NMR Studies on the Structure of Yeast Sis1 and the Dynamics of Its Interaction with Ssa1-EEVD

**DOI:** 10.3390/molecules30010011

**Published:** 2024-12-24

**Authors:** Carolina O. Matos, Glaucia M. S. Pinheiro, Icaro P. Caruso, Gisele C. Amorim, Fabio C. L. Almeida, Carlos H. I. Ramos

**Affiliations:** 1 Institute of Chemistry, University of Campinas UNICAMP, Campinas 13083-862, SP, Brazil; carol.omatos@gmail.com (C.O.M.); glauciasquizato.pinheiro@hotmail.com (G.M.S.P.); 2 Multiuser Center for Biomolecular Innovation (CMIB), Department of Physics, São Paulo State University (UNESP), São Jose do Rio Preto 01049-010, SP, Brazil; icaro.caruso@unesp.br; 3 National Center of Nuclear Magnetic Resonance (CNRMN), CENABIO, Federal University of Rio de Janeiro, Rio de Janeiro 21941-853, RJ, Brazil; gisele.amorim@caxias.ufrj.br; 4 Multidisciplinary Center for Research in Biology (NUMPEX-Bio), Campus Duque de Caxias Federal University of Rio de Janeiro, Duque de Caxias 25240-005, RJ, Brazil; 5 National Institute of Science and Technology for Bioimage and Structural Biology INBEB, Rio de Janeiro 21941-902, RJ, Brazil; 6 Institute of Medical Biochemistry, Federal University of Rio de Janeiro, Rio de Janeiro 21941-853, RJ, Brazil

**Keywords:** HSP40, Sis1, J-domain, protein–protein interaction, HSP70, NMR

## Abstract

HSP70 chaperones play pivotal roles in facilitating protein folding, refolding, and disaggregation through their binding and releasing activities. This intricate process is further supported by J-domain proteins (JDPs), also known as DNAJs or HSP40s, which can be categorized into classes A and B. In yeast, these classes are represented by Ydj1 and Sis1, respectively. While both classes stimulate the ATPase activity of Ssa1 (yeast HSP70) through the J-domain, only class B JDPs possess the unique ability to efficiently stimulate Ssa1 in disaggregation processes. The C-terminal EEVD motif of HSP70 plays a crucial role in mediating these interactions by connecting with both client proteins and JDPs. However, the removal of the EEVD motif disrupts the capacity of HSP70 to associate with class B JDPs, and the intricacies of the interaction between these two proteins remain incompletely understood. We employed NMR spectroscopy to investigate the structure and dynamics of the class B J domain protein (JDP) of *S. cerevisiae* (Sis1) complexed with an EEVD peptide of Ssa1. Our study is based on the extraordinary 70.5% residue assignment of the full-length (352 residues long) Sis1. Our findings revealed that EEVD binds to two distinct sites within the C-terminal domain I (CTDI) of Sis1, to the J domain and to the GF-rich loop located between the J domain and α-helix 6 (a structure identified by this work). We propose that the interaction between EEVD and Sis1 facilitates the dissociation of α-helix 6, promoting a conformational state that is more favorable for interaction with Ssa1. We also employed α-synuclein as a substrate to investigate the competitive nature between EEVD and the client protein. Our experimental findings provide evidence supporting the interaction of EEVD with the client protein at multiple sites and essential insights into the mechanistic cycle of class B JDPs.

## 1. Introduction

Chaperones from the 70 kDa heat shock protein (HSP70) superfamily play crucial roles in the cellular proteostasis system. These chaperones are composed of three main regions: a nucleotide-binding domain (NBD), a substrate-binding domain (SBD), and a disordered region that extends to the C-terminus, which ends with an EEVD motif [1,2,3]. The SBD further divides into two subdomains: the SBDβ, also known as the base subdomain, and the SBDα, referred to as the lid subdomain. The EEVD motif plays a significant role in both the intramolecular regulation of HSP70 function and intermolecular interactions with J-domain proteins (JDPs).

JDPs, also known as HSP40s or DNAJs, form a crucial family of cochaperones essential for facilitating the delivery of client proteins. Generally, these JDPs bind to the interface that links the NBD and the SBD of HSP70. This binding process is pivotal for activating and enhancing the ATPase activity of Hsp70 [4,5].

**Figure 1:**
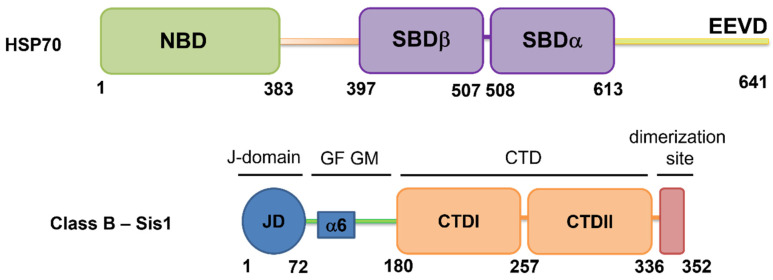
**Domain Architecture of HSP70 and Sis1.** Top: Schematic representation of HSP70, which is composed of a nucleotide-binding domain (NBD) shown in green and a substrate-binding domain (SBD) in purple, divided into SBDβ (base) and SBDα (lid) subdomains. These are connected by a flexible linker (depicted in orange) and a C-terminal disordered region that terminates in a highly conserved EEVD motif (shown in yellow). Bottom: Schematic representation of the domain organization of Sis1, a Class B JDP from yeast. The domains are as follows: JD: J-domain (blue, contains α-helices 1 to 5); GF: Gly-Phe-rich region; and GM: Gly-Met-rich region (green). The GF region of Sis1 contains α-helix 6, which is conserved in Class B JDPs and is described as α-helix 5 for DNAJB1 [11]; CTD: C-terminal domain (I and II; orange); and dimerization domain (red).

JDPs are classified into three distinct classes, each defined by the presence of a J-domain. In classes A and B, this domain is located at the N-terminal and is followed by a disordered region (IDR). In class A, this IDR is rich in glycine-phenylalanine (GF), whereas in class B, it consists of a GF region succeeded by a region rich in glycine-methionine (GM). Classes A and B have homologous C-terminal β-barrel domains, which are divided into CTDI and CTDII, as well as a dimerization domain [6,7,8,9]. Two cytosolic and dimeric JDPs from *Saccharomyces cerevisiae*, namely, Class A Ydj1 and Class B Sis1, are known to stimulate the ATPase activity of Ssa1 (yeast HSP70) and its function [10]. The architecture domains of HSP70 and JDP Class B Sis1 are shown in Figure 1.

Although both classes A and B stimulate the ATPase activity of HSP70 through the J domain, they exhibit distinct characteristics [12,13,14]. Notably, *sis1* is an essential gene, whereas *ydj1* is not. Knocking out the *ydj1* gene results in severe growth defects and reduced stress tolerance in cells. Conversely, the overexpression of Sis1 can partially mitigate this slow-growth phenotype. In contrast, Ydj1 overexpression does not restore the viability of cells in which the *sis1* gene is knocked out [11,12]. Ydj1 accounts for approximately 30% of the ability of Sis1 to bind Ssa1, as determined by ELISA. Notably, even when the G/F region and the CTDI are deleted from Sis1, 50% of the binding ability is retained [15].

The EEVD motif of yeast HSP70/Ssa1 can bind to Sis1 through electrostatic interactions, which is crucial for its chaperone activity [16]. Deletion of EEVD (HSP70_ΔEEVD_) disrupts the ability of Sis1 to associate with HSP70 but does not impact the interaction between Ydj1 and HSP70. When the J-domain of Ydj1 or Xdj1 (a paralog of Ydj1) is substituted for that of Sis1, the modified Sis1 associates with HSP70_ΔEEVD_. The interaction between Sis1 and EEVD is crucial for recruiting Ssa1 (*S. cerevisiae* HSP70) to bind to aggregated substrates [17]. Notably, only class B JDPs have the ability to effectively mediate HSP70 in fiber disaggregation [18], a process regulated in human DNAJAB1 through an autoinhibitory process involving the GF region [11]. While this process may be differently tuned in Sis 1 [14], specific details remain to be elucidated.

Our research group recently emphasized the crucial role of the transient interaction between Sis1 and HSP70 [19]. However, structural information regarding the interaction between JDPs and the EEVD motif remains limited, leaving several questions about the biological effects resulting from the association of these chaperones unanswered. This study provides a detailed NMR characterization of the dynamics and interaction between the J-domain of Sis1 (residues 1–81, herein referred to as Sis1_1–81_), the full-length Sis1 (residues 1–352, herein referred to as Sis1_1–352_), and the Ssa1–EEVD motif. Through this detailed analysis, we aim to provide valuable insights into the structural dynamics and functional implications of this crucial interaction, contributing to a deeper understanding of chaperone-mediated cellular processes.

## 2. Results and Discussion

### 2.1. The Structure of Sis1_1–81_:EEVD-Bound

**Figure 2:**
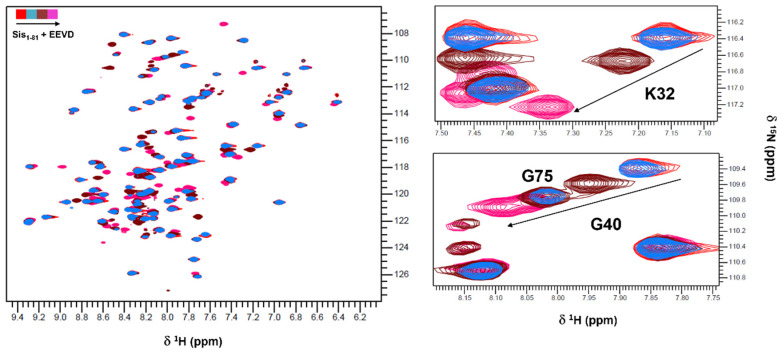
**Overlay of 2D ^1^H,^15^N HSQC spectra collected for ^15^N-Sis1_1–81_ at increasing EEVD peptide concentrations**. Sis1_1–81_ free (in red), Sis1_1–81_ + 1 mM EEVD-peptide (in blue), Sis1_1–81_ + 2 mM EEVD-peptide (in brown), and Sis1_1–81_ + 4 mM EEVD-peptide (in magenta); Sis1_1–81_ concentration was 1 mM in all conditions. The (**left side**) of the figure displays the full spectrum, whereas the (**right side**) represents the differential chemical shift data for residues K32 and G40 of Sis1_1–81_. The residue G75 was not perturbed by EEVD and is between the cross-peak of G40 at 2 (brown) and 4 mM (magenta). These residues are notably involved in binding with the EEVD peptide.

The interaction between a GPTIEEVD peptide, which represents the EEVD motif, and the J-domain was extensively explored by examining the structure of the complex formed between this motif and the isolated J-domain of Sis1. To achieve this goal, we utilized the Sis1_1–81_ construct, whose structure was previously determined by NMR [19]. This construct encompasses the J-domain (residues 1–72) and part of the GF region (residues 73–81). The investigation was initiated by titrating Sis1_1–81_ with the EEVD peptide, followed by a series of ^1^H-^15^N HSQC measurements (Figure 2). The resulting ^1^H-^15^N HSQC spectrum, representing the Sis1_1–81_:EEVD-bound complex, exhibited characteristics indicative of a well-folded and monomeric protein.

The subsequent experimental procedures included a set of standard triple resonance experiments to achieve precise residue assignments. We employed ^15^N- and ^13^C-edited NOESY experiments, which yielded 2750 intramolecular NOE (Nuclear Overhauser effect). This dataset was deemed sufficient to generate the folded structure of Sis1_1–81_ (for detailed statistics, please refer to Table S1). To calculate the structure of the Sis1_1–81_:EEVD-bound complex, we utilized ^13^C-edited half-filtered experiments in a sample containing the ^12^C-EEVD peptide and ^13^C-Sis1_1–81_. These experiments aimed to obtain unambiguous information on intermolecular NOEs. However, owing to the transient, low-affinity nature of the interaction, the NOESY spectra resulted in only a small number of intermolecular NOEs, which was insufficient for calculating the NOE-based structure of the complex. The measurement of the affinity constant (K_D_) was hindered by the limited solubility of the peptide, as its affinity lies within the millimolar range.

**Figure 3:**
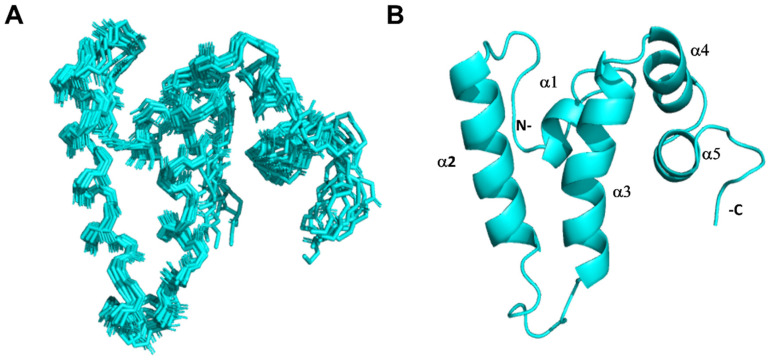
**The solution structure of Sis1_1–81_ bound to the EEVD peptide.** (**A**) An ensemble of the 20 lowest-energy structures of the J-domain of Sis1 (residues 1–81) with the HSP70 C-terminal peptide GPTIEEVD (Sis1_1–81_:EEVD-bound conformation). (**B**) A ribbon representation of the lowest-energy structure of the Sis1_1–81_:EEVD-bound conformation. The N- and C-termini, along with the α-helices (α1 to α5), are highlighted in the structure.

Upon structural calculation via Aria2.3/CNS1.21, we obtained a well-defined ensemble of 20 structures for Sis1_1–81_:EEVD-bound (Figure 3A). The bound conformation of Sis1_1–81_ consists of five α-helices (α1 to α5, as shown in Figure 3B), with four located in the J-domain (α1 to α4) and one at the boundaries with the GF region (α5). The global folded conformations of α1 (residues 6–11), α2 (19–33), α3 (42–56), α4 (58–66), and α5 (69–74) are similar to those found in free Sis1_1–81_ [17,19]. Specifically, α2 and α3 are antiparallel to each other and linked by a loop containing the conserved HPD motif (^34^His-Pro-Asp^36^) [20,21], which is consistent with the structural features observed in the free Sis1_1–81_ state. For comparison purposes, the human Sis1 homolog DNAJB1 features four helices (named I to IV) within its J-domain [11].

### 2.2. An EEVD Binding Site Is Identifiable in the J-Domain of Sis1

**Figure 4:**
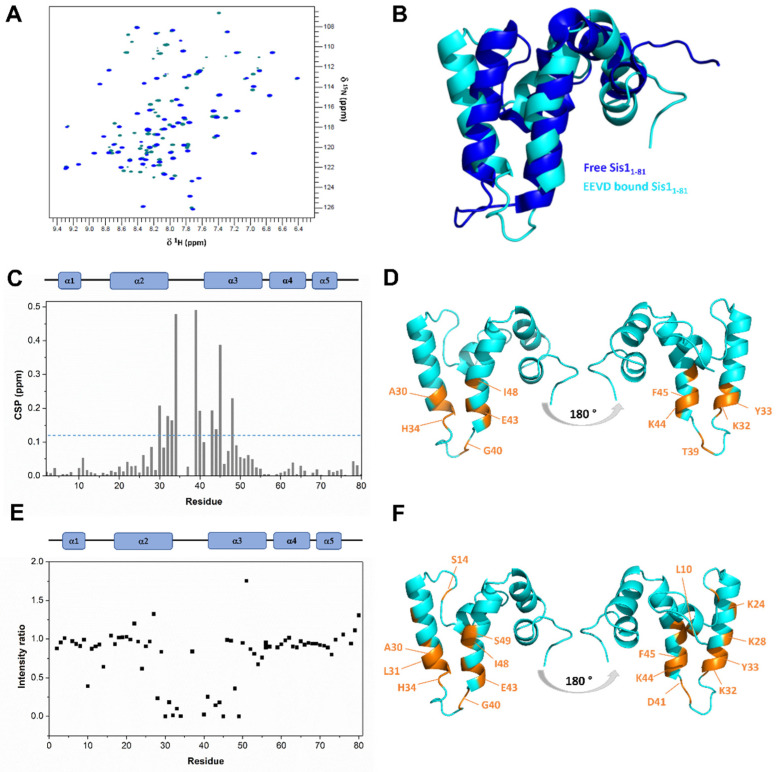
**NMR experiments revealed detailed interactions of EEVD peptide binding between α2 and α3 of Sis1.** (**A**) An overlay of 2D ^1^H-^15^N HSQC spectra is displayed, showing free Sis1_1–81_ (in blue) and EEVD-bound Sis1_1–81_ (4-fold molar excess; in cyan). (**B**) A superposition of the NMR structures of free Sis1_1–81_ (in blue, PDB 6D6X) and Sis1_1–81_:EEVD-bound (in cyan, from this work) is provided. (**C**) The chemical shift perturbation (CSP) of Sis1_1–81_ resonances, induced by the addition of the EEVD peptide, is illustrated. The CSP index is represented as vertical bars for each residue. Significant changes are defined as those with a CSP larger than the average plus one standard deviation (depicted by the blue dotted line in (**C**) and highlighted in orange in (**D**)). (**D**) CSPs are mapped (in orange) onto the Sis1_1–81_:EEVD-bound conformation. (**E**) Experimental intensity ratios of the backbone amide resonances in Sis1_1–81_ complexed with the paramagnetic EEVD peptide are presented. An intensity ratio of 1 indicated that the spin label had no effect on an amide proton. (**F**) PREs are mapped (orange) onto the Sis1_1–81_:EEVD-bound conformation.

The ^1^H-^15^N CSP of Sis1_1–81_ upon the addition of the EEVD peptide (at a 1:4 molar ratio) is shown in Figure 4A. Residues exhibiting significant chemical shift differences between the free and bound conformations were identified, indicating their direct or indirect association with the binding site. Several Sis1_1–81_ residues displayed substantial chemical shift variations in their ^1^H-^15^N-HSQC NMR spectra upon EEVD-peptide binding, delineating a well-defined region situated at the α2/α3 open patch in the J-domain. This interaction occurs in the fast exchange regime on the NMR time scale. In the bound state, the α2/α3 binding patch is more open than in the free state. There are two available structures in the free state, one in solution [19] and the other crystallographic [17]. Both are very similar and exhibit the α2/α3 binding patch in a closed conformation. Additionally, α3 becomes slightly twisted in the bound conformation, likely accommodating the EEVD peptide (Figure 4B). The RMSD between the free and bound Sis1_1–81_ states is 3.22 Å, as measured on Cα atoms. Residues A30, K32, and Y33 in α2, H34 and G40 in the loop between α2 and α3 and E43, K44, F45, and I48 in α3 presented the largest CSP values (Figure 4C). These findings indicate that these residues are either directly involved in contacts or indirectly affected by conformational changes induced by EEVD-peptide binding (Figure 4D). A comparison of the CSPs of EEVD-bound Sis1_1–81_ to those upon the addition of HSP70, as previously reported by our group [19], revealed notable similarities. Specifically, residues A29 in α2, H34, and T39 in the loop, F45 and F52 in α3, and Y67 in α4 were perturbed by either HSP70 or the EEVD peptide, suggesting that the results with the peptide mirror those with the full-length HSP70.

The main difference between the EEVD-bound conformation and the free Sis1_1–81_ lies in the N-terminal portion of α2 and the C-terminal portion of α3. This difference is significant for several reasons: (i) the EEVD-bound conformation is based on a large number of intramolecular NOEs (2750), yielding a well-defined structure (Figure 3); (ii) there are two available structures for the free state [17,19], both showing a proximity of α2 and α3 stabilized by hydrophobic interactions between L22, L55, and the aliphatic chain of R61; (iii) this hydrophobic interaction is altered in the EEVD-bound conformation, with L22 close to F52 and R61 moving farther apart; and (iv) many NOEs support these conformational changes. Specifically, there is an NOE connecting L22. Hγ in the solution structure of the free protein that is missing in the EEVD-bound protein.

We also employed paramagnetic relaxation enhancement (PRE) on Sis1_1–81_ residues induced by an N-terminal TEMPO-labeled EEVD peptide to advance our understanding of the impact of EEVD binding on Sis1_1–81_. Distance restraints from the EEVD peptide to the protein were measured by analyzing intermolecular PRE rates for the backbone amide groups of Sis1_1–81_ in complex with spin-labeled EEVD (TEMPO-EEVD) (Figure 4E; see also Materials and Methods). Residues L10 in α1; S14 in the loop between α1 and α2; K24, K28, A30, L31, K32, and Y33 in α2; H34, G40, and D41 in the loop between α2 and α3; and E43, K44, F45, I48, and S49 in α3 presented the largest CSP values (Figure 4F).

The CSP and PRE for the HPD triad are missing (Figure 4C,E). This is due to the lack of assignment for P35 and K36 NH in Sis1_1–81_ in the absence of EEVD [22] (BMRB #27405). The lack of assignment of the K36 NH is due to extreme line broadening caused by conformational exchange. Interestingly, when Sis1_1–81_ is bound to EEVD, we were able to almost fully assign P35 and K36, including K36 NH. This finding indicates the stabilization of a conformational state when Sis1_1–81_ is bound to the EEVD peptide, providing further evidence of binding. The lack of CSP and PRE information for the prolines (P35 and P38) is due to the absence of an NH. For residue K37, we observed a low CSP and PRE. K37 is at the top of the α2/α3 loop and likely remains dynamic in the bound conformation. Notably, Sis1_1–81_ exhibited CSP and PRE values in the same region between α2 and α3, suggesting conformational selection upon EEVD peptide binding.

### 2.3. Molecular Dynamics Simulations Reveal the Binding of the EEVD Peptide Between α2 and α3 of Sis1

**Figure 5:**
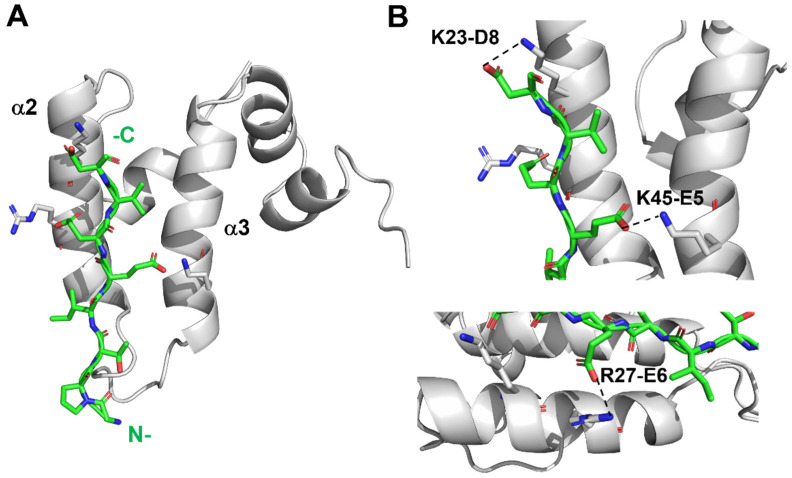
**Structure of the Sis1_1–81_:EEVD-peptide complex.** (**A**) The structure of the Sis1_1–81_:EEVD-peptide complex is depicted through a ribbon representation, showing the lowest-energy structure derived from HADDOCK via an NMR data-based model. In this complex, the peptide binds in an extended manner between helices α2 and α3, positioning its N-terminal region near the HPD loop. Sis1_1–81_ is represented in gray, whereas the EEVD peptide is depicted as sticks, with carbon in green, oxygen in red, and nitrogen in blue. (**B**) A detailed view of the intermolecular salt bridges (K45-E5, K23-D8, and R27-E6) formed between Sis1_1–81_ and the EEVD peptide. The corresponding structural details are available in PDB entry 8EOD.

PRE and CSP data, in conjunction with HADDOCK [23], were employed to generate NMR data-based structural models for the Sis1_1–81_:EEVD-peptide complex (refer to Figure 5 for the lowest energy structural model; further details on the methodology and model construction are provided in the Supplementary Material: PART 2: Molecular Docking and Molecular Dynamic (MD) Simulations). The docking process involved the presentation of ten clusters, with the best-docked cluster selected based on a low Haddock score and a low root mean square deviation (RMSD) value (see Table S2).

Molecular dynamics (MD) simulations further contributed to the generation of the Sis1_1–81_:EEVD-peptide complex, and the RMSD values of the backbone atoms of Sis1_1–81_ and the EEVD peptide were assessed from the starting structure (HADDOCK model, Figure 5). In the structural model of the Sis1_1–81_:EEVD-peptide complex, the peptide assumes an extended orientation between α2 and α3, positioning its N-terminus in proximity to the HPD loop (Figure 5). This structural arrangement aligns well with both the CSP and the PRE data, confirming the accuracy of the model.

**Figure 6:**
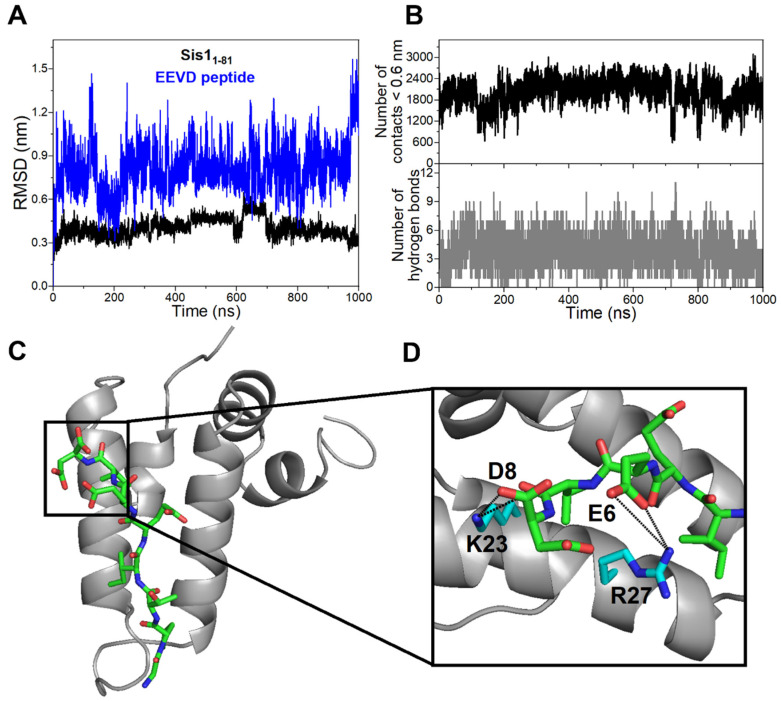
**Analysis of parameters from 1.0 μs molecular dynamics simulations of the Sis1_1–81_:EEVD peptide complex.** (**A**) The RMSD values of the backbone atoms of Sis1_1–81_ (depicted in black) and the EEVD peptide (in blue) from the starting structure (docking structure, refer to Figure 5) are illustrated. (**B**) The number of contacts formed between atoms (<0.6 nm, top) and the number of hydrogen bonds (bottom) involving Sis1_1–81_ and the EEVD peptide are displayed. (**C**) A representative structure of the Sis1_1–81_:EEVD complex, obtained from cluster analysis of the trajectory, is depicted. Sis1_1–81_ is represented as gray cartoons, and the EEVD peptide is shown as sticks, with carbon in green, oxygen in red, and nitrogen in blue. (**D**) A detailed view of the intermolecular salt bridges (K23-D8 and R27-E6) formed between Sis1_1–81_ and the EEVD peptide is provided.

The RMSD analysis indicated stable behavior for Sis1_1–81_ throughout the 1 μs simulation. Moreover, the RMSD of the EEVD peptide slightly increased during the initial 20 ns and stabilized thereafter throughout the entire MD simulation (Figure 6A). The average RMSD values were 4.0 ± 0.1 Å and 8.1 ± 0.2 Å for Sis1_1–81_ and the EEVD peptide, respectively (Figure 6B). Evaluation of the number of contacts (distance < 0.6 nm) formed between atoms of Sis1_1–81_ and the EEVD peptide revealed continuous interactions throughout all the MD simulations, averaging 2027 ± 339 contacts (Figure 6B, top). The structural model indicated the formation of the Sis1_1–81_:EEVD peptide, with an average of 4 (3.7 ± 1.5) intermolecular hydrogen bonds persisting throughout all the MD simulations (see Figure 6B, bottom). Residues Q20-V7, K23-D8, R27-E6, H34, and E6-Y26 exhibited percentages of persistence greater than 10% (see Table S3).

The structural model also confirmed the extended conformation of the EEVD peptide between α2 and α3, which aligns well with the NMR and HADDOCK data (Figure 6C). Notably, two salt bridges were observed between K23-D8 and R27-E6 (Figure 6D, Table S3). The importance of these salt bridges is underscored by the conservation of the i, i + 4 charged residue arrangement, with residues K23 and R27 being 4 residues apart (i, i + 4). This arrangement is conserved in other DNAJB models (DNAJB1 and DNAJB6) and in some tetratricopeptide (TPR) domains. Taken together, these examples suggest a Sis1_1–81_:EEVD-peptide interaction through similar salt bridges. The TPR motif, characterized by a helix-loop-helix structure similar to J-domain α2, loop, and α3, further supports the idea that these elements interact via analogous arrangements [24] (a more in-depth discussion on this topic is provided later).

### 2.4. Insights into the Interaction Between the J-Domain of Sis1 and the EEVD Peptide

**Figure 7:**
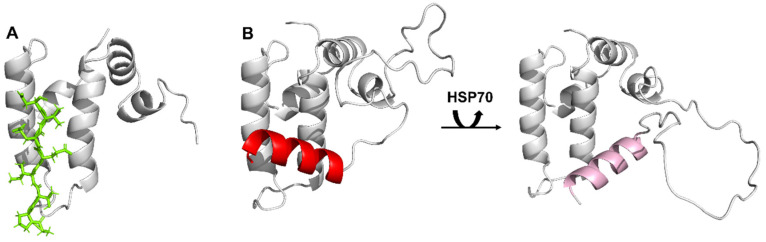
**Insights into the interaction between the J-domain of Sis1 and Ssa1.** (**A**) The EEVD motif (highlighted in green) adopts an extended orientation between α2 and α3 (shown in gray), positioning its N-terminus in close proximity to the HPD loop. (**B**) Illustrative representation of residues 1–81 of Sis1, encompassing the J-domain and a portion of the G/F domain, as predicted by AlphaFold2 (left), showing how α-helix 6 blocks the EEVD binding site. On the right, a representation shows the alleviation of this autoinhibition upon interaction with HSP70. The region between helices 2 and 3 of the J-domain is obstructed by α-helix 6 (shown in red or pink) located in the G/F region. This autoinhibition is relieved through interaction with the EEVD motif of HSP70 [11].

One of the objectives of this study was to investigate the interaction between the EEVD motif and the J-domain of the yeast class B JDP Sis1. Our NMR characterization successfully identified an EEVD site in the J domain of Sis1, as illustrated in Figure 7A. This discovery is important because the C-terminal tails of Ssa1 play crucial roles in facilitating proper protein folding in Sis1, unlike in class A Ydj1 [17]. Initial indications of Sis1 binding to the EEVD motif were first reported by Yu et al. [17]. Their study demonstrated that the deletion of EEVD (Hsp70_∆EEVD_) resulted in the loss of the ability of Sis1 to collaborate with HSP70 in protein refolding. Moreover, they revealed that the disruption of a single residue in the J-domain of Sis1, leading to the disruption of a salt bridge in this domain, or the complete replacement of the J-domain with those of Ydj1 or Xdj1 (a Ydj1 paralog), restored the ability to partner with Hsp70_∆EEVD_ in refolding.

To understand the binding of the EEVD peptide, full-length Sis1 (Sis1_1–352_) was also investigated (see also the following sections). The analysis of both the CSP and PRE data revealed interactions with residues of the J-domain (10, 14, and 42) and the GF region, mainly residues 78 to 106, which are N-terminal to α-helix 6. Notably, the region between α2 and α3 of the J-domain is obstructed by another helix located in the G/F region (usually named helix 5 or the inhibitory helix in human DNAJBs; Figure 7B), as demonstrated by NMR studies conducted on human JDPs DNAJB1 [11], DNAJB6 [25], and DNAJB8 [26]. This hindrance is identified as an autoinhibition mechanism, given the presence of an Hsp70-interacting surface on the α2/α3 hairpin. Removal or destabilization of the inhibitory helix eliminates competition with HSP70. According to these studies, the EEVD interaction serves to alleviate the autoinhibition of the J-domain by the G/F linker (see Figure 7B). This hypothesis is supported by our results indicating that this blockage could affect EEVD binding to the J-domain, but no contacts between EEVD and the J-domain have been detected in DNAJB1 [11]. However, deletion of the inhibitory helix in DNAJB1 fully restored luciferase disaggregation activity with Hsp70_ΔEEVD_ [11], whereas deletion of the equivalent autoinhibitory helix in Sis1ΔH5 had no similar effect [14], suggesting that the regulation of J-domain inhibition might differ between these two orthologs. This discrepancy implies that the additional interaction of the J-domain of Sis1 with the EEVD motif likely expands the network of possible interactions within this chaperone complex. Moreover, it is crucial to consider our results, which demonstrate that the binding of EEVD to the J domain induces the opening of α2 and α3. This effect is likely to influence the interaction of these helices with the inhibitory helix (see Figure 7B). Importantly, these findings align with results from other groups, collectively supporting the notion that HSP70-EEVD plays a significant role in relieving the autoinhibitory effect. Regardless, further studies may reveal novel mechanisms for cooperation between Hsp70 and class B JDPs.

The highly conserved EEVD motif at the C-terminus of HSP70 is well established as the sequence that binds to the concave face of the TPR domains present in TPR-containing cochaperones. A TPR motif is characterized by a pair of antiparallel α-helices connected by a short turn or loop, resembling the α2/α3 arrangement in the J-domain (see Figure 6C). In our study, we observed two salt bridges between helices in the J-domain and the EEVD peptide: K23-D8 and R27-E6 (as shown in Figure 6D). The significance of these salt bridges is highlighted by the conservation of the i, i + 4 charged residue arrangement, where residues K23 and R27 are positioned 4 residues apart (i, i + 4). Notably, this charged residue arrangement (i, i + 4) is present in certain tetratricopeptide (TPR) domains, stabilizing positions D(0) (D8) and E(-2) (E6) in the EEVD motifs, as observed in the TPRs of Tom71, SGT2, and FKBP92–380, among others. These instances suggest a Sis1_1–81_:EEVD-peptide interaction involving similar salt bridges. Furthermore, the TPR motif, characterized by a helix-loop-helix structure similar to the J-domain α2, loop, and α3, supports the idea that these elements interact through analogous arrangements [24]. A TPR-like binding mechanism might offer insights into the diverse affinities displayed by different J domains for the EEVD motif. This mirrors the variability in affinity observed among cochaperones containing TPR motifs [27].

### 2.5. The Interdomain Dynamics of Sis1_1–352_ Are Affected by EEVD

Using the preeminent 70.5% residue assignment of full-length Sis1 (352 residues), we first examined the dynamics of the free protein and the changes triggered by its interaction with the EEVD peptide. The ^15^N longitudinal (R_1_) and transverse (R_2_) relaxation rates of Sis1_1–352_ in both the free and EEVD-bound states were measured. Table S4 summarizes the ^15^N R_2_/R_1_ ratios and the apparent rotational diffusion ($\tau_{c}^{app}$) of each domain within the full-length protein context [28]. $\tau_{c}^{app}$ reflects the rotational freedom of each domain, both in the free and EEVD-bound states, and was calculated by excluding residues in conformational exchange or with thermal flexibility, considering only values within one standard deviation (SD) of the mean.

**Figure 8:**
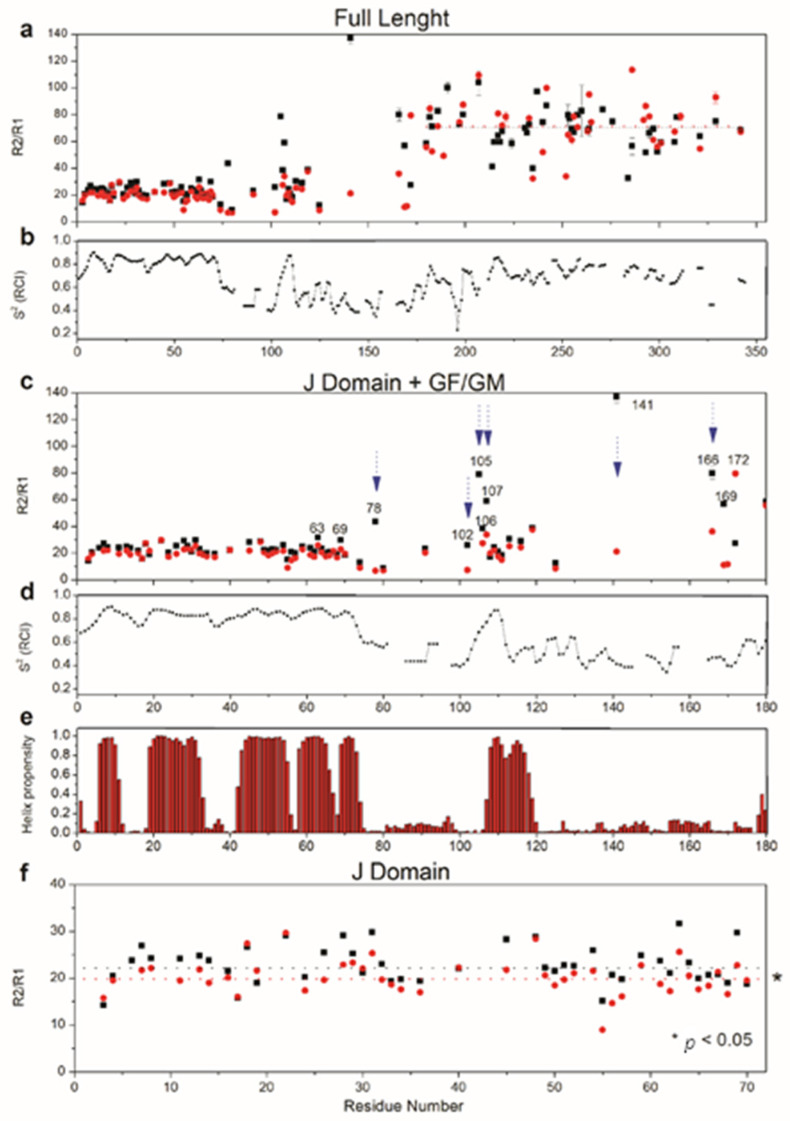
Relaxation parameters, chemical shift-based order parameters, and Talos-N secondary structure predictions for Sis1_1–352_. (**a**) ^15^N-R_2_/R_1_ for Sis1_1–352_ (full-length), shown in the free state (black squares) and bound to EEVD (red circles) for each residue. The dotted lines indicate the average ^15^N-R_2_/R_1_ values for the CTDI, CTDII, and dimerization domains. (**b**) Chemical shift-based order parameter (S^2^) for residues in free Sis1_1–352_. The lower the value of S^2^ is, the greater the internal flexibility. (**c**) Zoomed plot (residues 1 to 180) showing ^15^N-R_2_/R_1_ for the J-domain and GF/GM regions for the free state (black squares) and the EEVD-bound state of Sis1_1–352_ (red circles). The blue arrows highlight residues where ^15^N-R_2_/R_1_ decreased upon EEVD binding. (**d**) Zoomed plot (residues 1 to 180) showing S^2^ values for the J-domain and GF/GM regions in free Sis1_1–352_. (**e**) Zoomed plot (residues 1 to 180) showing the helical propensity predicted by Talos-N for the J-domain and GF/GM region in free Sis1_1–352_. (**f**) Zoomed plot (residues 1 to 72) showing ^15^N-R_2_/R_1_ values for the J-domain in the free state (black squares) and EEVD-bound state (red circles). The dotted lines indicate the average ^15^N-R_2_/R_1_ values. *, statistically significant (*p* < 0.05) decreases in average ^15^N-R_2_/R_1_ values.

The relaxation parameters for free *Sis1*_1–352_ (Figure 8a, Table S4) revealed notable differences in the dynamics of the J-domain (residues 1–72), GF (73–121), GM (122–178), CTDI (179–257), CTD II (258–335) and dimerization (DD, 336–352) domains. Significant differences in the ^15^N R_2_/R_1_ ratios were observed for the J-domain (22 ± 2), GF/GM (30 ± 13), and CTD (70 ± 8), indicating that the domains located at the C-terminus, CTD, and DD have the most restricted rotational freedom. The calculated apparent rotational correlation time ($\tau_{c}^{app}$) indicated that the domains had greater rotational freedom than expected for a rigid globular protein of 75.2 kDa, reflecting the anisotropic, scissor-like structure of *Sis1*_1–352_. The CTD exhibited the most restricted domain motion ($\tau_{c}^{app}$ = 18 ± 2 ns), likely due to its proximity to the dimerization domain. In contrast, the J-domain and GF/GM regions behaved as highly flexible arms, with $\tau_{c}^{app}$ values of 9.8 ± 1.0 ns and 11.0 ± 5.0 ns, respectively. Notably, the ^15^N R_2_/R_1_ values of the GF/GM region (sd = 5) were greater than those of the J domain (sd = 1) or CTD (sd = 2).

Chemical shift-based order parameter (S^2^) analysis for Sis1_1–352_ (Figure 8b) and Sis1_1–180_ (Figure 8d), obtained via TALOS-N [29,30], provided insight into the structural order of each region or domain. We observed high order for the well-folded J-domain, CTDI, CTDII, and dimerization domain (DD). In contrast, the GF/GM exhibited high backbone flexibility, which is characteristic of an IDR, except for residues 104–112, which are highly ordered. Region 104–112 contains the N-terminal portion of α-helix 6, spanning residues 107–119 (Figure 8e). While the higher order in the helical segment was expected, the mismatch between the ordered residues within the GF (104–112, Figure 8d) and α-helix 6 (107–119, Figure 8e) was surprising. The observed order of residues 104–112 of the α-helix 6 (Figure 8b) supports its interaction with the J-domain. However, the mismatch between the ordered (104–112) and helical residues (107–119) suggests an equilibrium between a J-domain-associated (on-state) and a free or J-domain-dissociated (off-state) α-helix 6. In solution, the chemical shift likely reflects the average between these two states, as observed for DNAJB1 [11] and DNAJB6 [25]. Additionally, the lower structural order of the C-terminal portion of α-helix 6 (residues 113–119) may indicate partial or total unfolding of the helix in the off-state. This finding is consistent with secondary structure predictions for Jpred4 [31], which do not predict a well-folded α-helix 6 in the off-state, indicating that it would be disordered. Further evidence of an on-off equilibrium is the conformational exchange for residues S78, G102, A105, F106, and S107 in the GF region and N-terminal to α-helix 6, as indicated by their high ^15^N R_2_/R_1_ values (Figure 8c).

The presence of EEVD led to a subtle but significant increase in the rotational freedom of the J-domain. $\tau_{c}^{app}$ decreased from 9.8 ± 1.0 ns to 9.2 ± 1.0 ns, which was statistically significant (*p* < 0.05, Figure 8f, Table S4) given the large number of measurements (35 measurements of ^15^N R_2_/R_1_ in the absence and 37 in the presence of the EEVD). Most ^15^N R_2_/R_1_ values decreased in the presence of EEVD (Figure 8f), and this decrease for residues S78, G102, A105, F106, and S107 (Figure 8a,c) suggests that the on-off equilibrium of α-helix 6 was suppressed by EEVD binding. Additionally, significant decreases in ^15^N R_2_/R_1_ were observed for residues G141, G166, and S169 (Figure 8a,c), suggesting that EEVD binding quenched the conformational exchange in these GM region residues. The only residue with an increase in ^15^N R_2_/R_1_ upon EEVD binding was S172. However, no significant change in $\tau_{c}^{app}$ for the CTD was observed in the presence of EEVD. These results indicate that EEVD binding induces significant changes in the dynamics of Sis1_1–352_.

### 2.6. EEVD Interacts at Multiple Sites Within Sis1_1–352_

**Figure 9:**
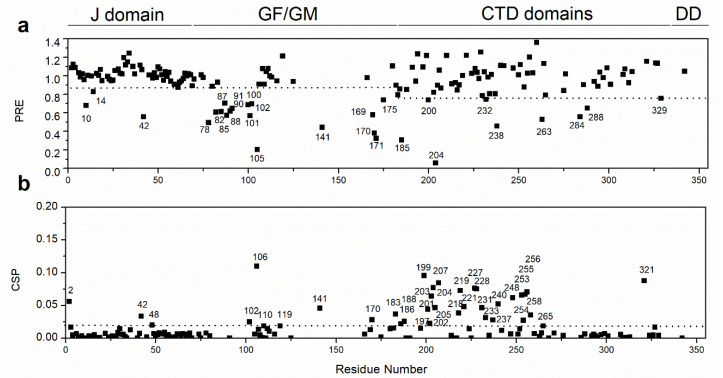
**EEVD binding to full-length Sis1 (Sis1_1–352_).** (**a**) Experimental intensity ratios of backbone amide protons in full-length Sis1_1–352_ in complex with an EEVD paramagnetic peptide (see also Materials and Methods) are shown for each residue. An intensity ratio of 1 indicates that the spin label has no effect on the amide proton. Residues with significant PRE effects are indicated. PRE represents the normalized intensity ratio ($I^{para}/I^{dia}$) between the paramagnetic and diamagnetic (reduced with ascorbate) TROSY spectra. (**b**) Chemical shift perturbation (CSP) of Sis1_1–352_ resonances upon the addition of the EEVD peptide. CSP values greater than the mean plus one standard deviation are marked by dotted lines.

**Figure 10:**
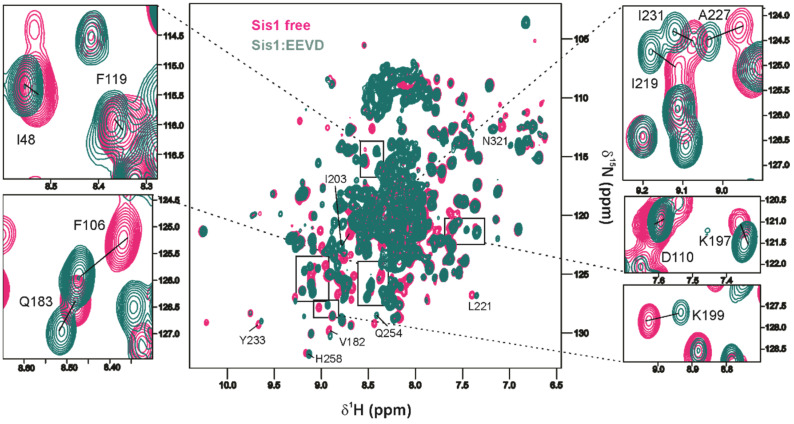
**Overlay of ^1^H,^15^N TROSY HSQC spectra for full-length Sis1 (^15^N-Sis1_1–352_) with an excess of EEVD peptide (1:4 ratio)**. The spectra for Sis1_1–352_ in the absence (magenta) and presence (green) of the EEVD peptide are shown. The full spectra, shown in the middle, highlight residues I48 (J-domain); F106, D110, and F119 (GF region); and Q183, K197, K199, I219, A227, and I231 (CTDI), all of which exhibit perturbations in the presence of EEVD, indicating their involvement in the interaction.

To further investigate the effects of EEVD binding to Sis1_1–352_, we used two methods to map the binding sites. Paramagnetic relaxation enhancement (PRE) of the Sis1_1–352_ residues is elicited by an N-terminal TEMPO-labeled EEVD peptide (Figure 9a), and chemical shift perturbation (CSP) is caused by EEVD peptide binding (Figure 9b and Figure 10). We successfully assigned more than 70% of the backbone of Sis1_1–352_, and to ensure greater accuracy, all overlapping peaks were excluded from the analysis. We identified two major regions of interaction, guided by residues with PRE < 0.88 (1–182) or 0.7 (183–352) (dotted lines in Figure 9a) or CSP > 0.018 (dotted line in Figure 9b): (i) the GF IDR, spanning residues 78–102, located between the J-domain and α-helix 6, and (ii) part of the GM region and the CTDI domain (residues 180–256). Notably, both regions in free Sis1_1–352_ contain residues undergoing conformational exchange, as indicated by their above-average ^15^N-R_2_/R_1_ values (Figure 8a). Upon EEVD binding, ^15^N-R_2_/R_1_ returned to average values, suggesting that the millisecond to microsecond conformational motions were suppressed by the interaction.

The observed PRE and CSP across multiple regions of the protein suggest that EEVD binds to more than one site, predominantly the GF and CTDI regions. Specifically, the PRE and CSP effects observed in the J-domain, GF, and CTDI, as well as in a few residues of the GM region just before the CTDI (Figure 8), support this hypothesis. CSPs reflect changes in the chemical environment caused by the presence of a ligand, which may result from direct or indirect interactions with the ligand. In contrast, PRE provides a direct measure of distances between the ligand and the protein, with PRE broadening the NMR signal of any nuclear spin within approximately 30 Å of the paramagnetic center [32]. The PRE effect, indicated by a significant decrease in $I^{para}/I^{dia}$, confirmed the direct interaction of EEVD at multiple sites, corroborating the CSP findings. For the J domain, we observed both CSP and PRE: V2 (CSP), A10 (PRE), S14 (PRE), T42 (PRE and CSP), and I48 (CSP). For the GF domain, we also observed CSP and PRE: S78, G82, G85, A87, G88, G90, A91, F100, S101, G102, and A105 (CSP) and G102, F106, D110, and F119 (PRE). Interestingly, the PRE effect was observed in the N-terminal vicinity of α-helix 6 (G102 and F106) and at α-helix 6 (D110, exposed to the solvent, and F119, facing the J domain interacting patch, α2-loop-α3). The weaker PRE and CSP observed for the J domain compared to those observed in the CTDI can be attributed to competition between the EEVD motif and α-helix 6, which is present only in the full-length SIS1.

For CTDI and the GM region, just before CTDI, we observed many residues with CSP and PRE. We observed the PRE for the following residues: S169, A170, S171, and T175 are at the GM pre-CTDI, and N185, S200, G204, I232, and Q238 are at the CTDI. We observed CSP for the following residues: A170 (GM, pre-CTDI), Q183, L186, V188, K197, K199, F201, K202, I203, G204, R205, D218, I219, L221, A227, G228, I231, K233, G237, D238, N240, T248, I253, Q254, E255, K256, H258, and G265 (CTDI). Finally, NMR data-derived docking of the EEVD peptide to Sis1_1–352_ supported the multiple binding site hypothesis.

### 2.7. Structural Models for the Interaction of EEVD with Sis1_1–352_

**Figure 11:**
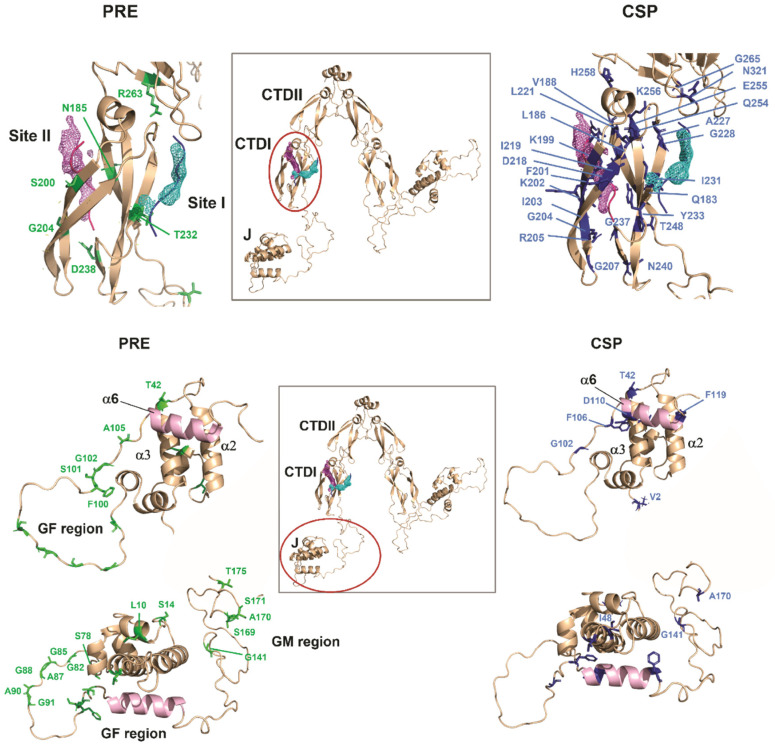
**TOP: EEVD binds to multiple sites on the CTDI of Sis1_1–352_.** An illustrative representation of the Sis1 structure, predicted by AlphaFold, highlights the CTDI, showing the atomic probability density map of the EEVD peptide at sites I (cyan) and II (magenta). Residues identified by PRE (**left**) and CSP (**right**) are labeled in green and blue, respectively. Site II is composed of the β1, α, and β2 motifs, whereas site I involves β4. **BOTTOM: EEVD binds to multiple sites in the J domain, GF, and GM regions of Sis1_1–352_.** A cartoon representation of the Sis1 structure, as predicted by AlphaFold, highlights the J domain and the GF region. Residues mapped by PRE (**left**) and CSP (**right**) are labeled in green and blue, respectively. The α-helix 6, shown in green, is predicted to adopt an autoinhibited conformation, blocking the J domain binding patch. Notably, residues T42 and I48, located in the α-helix 3, exhibit either PRE or CSP effects, suggesting the involvement of the J domain in the interaction with EEVD.

Haddock [24] was used to dock the EEVD peptide into various regions of Sis1_1–352_ (further details on the methodology and model construction are provided in the Supplementary Material: PART 2: Molecular docking and molecular dynamics (MD) simulations). The initial docking targeted the dimeric CTDI (180–257), using only the PRE and CSP data specific to this region. The results revealed that the EEVD peptide binds to the CTDI at two primary interaction sites, named sites I and II, which are located on opposite faces of the domain (Figure 11 and Table 1). The atomic probability density maps for site I (cluster I) and site II (cluster II) are described by the meshes in cyan (site I) and magenta (site II) in Figure 11. The position of the density maps matches the experimental PRE and CSP in the CTDI, as measured for Sis1_1–352_ in the presence of the EEVD peptide. For site I, the density map also coincides exactly with the position of an HSP70-EEVD peptide complexed with human DNAJB1 [33], and one of the poses even forms an antiparallel β-strand with the CTDI β-strand 4 (β4), the same configuration of the EEVD complex at site I of human DNAB1 [33]. For site II, the density map is between β1 and β2, while both crystal structures describe the interaction of the EEVD peptide with either DNAB1 (3AGY) or Sis1 (2B26), which interact with β2, forming an antiparallel β-strand.

### 2.8. The EEVD Peptide Competes for the Binding Site of a Client Protein

**Figure 12:**
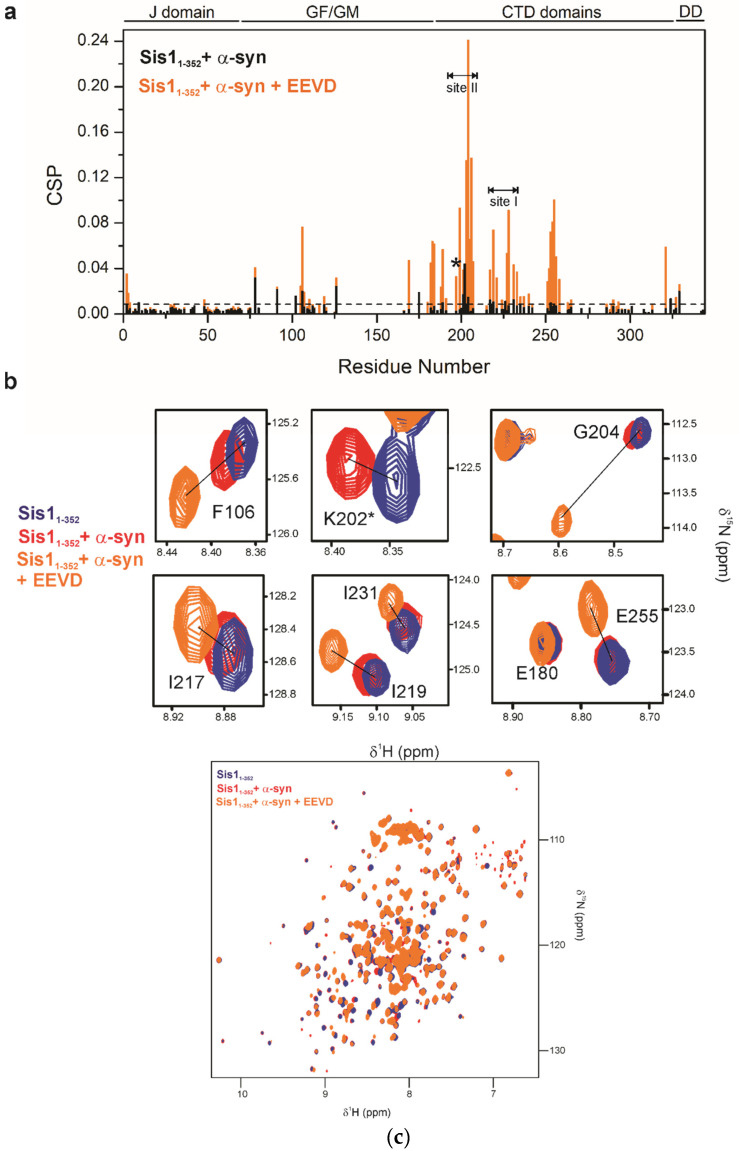
**Client protein and EEVD peptide compete for the same sites on Sis1_1–352_.** (**a**) Chemical shift perturbation (CSP) analysis of Sis1_1–352_ with α-synuclein (α-syn, black) and Sis1_1–352_ with the α-syn + EEVD peptide (orange). (**b**) ^1^H-^15^N TROSY HSQC zoom spectra of free Sis1_1–352_ (blue) showing CSP upon interaction with α-syn (red) and α-syn + EEVD (orange). Residues involved in competition for the same binding sites are indicated. Sis1_1–352_ can simultaneously interact with both the client α-syn peptide and the HSP70-EEVD peptide. An asterisk (*) indicates that the resonance for residue K202 disappeared upon the addition of the EEVD peptide, likely due to line broadening caused by a conformational exchange. (**c**) Overlay of 2D ^1^H,^15^N TROSY HSQC spectra collected for ^2^H-^15^N-Sis1_1–352_ with the α-syn and EEVD peptides. Full spectra of free Sis1_1–352_ (blue), Sis1_1–352_ interacting with α-syn (red), and α-syn+ EEVD (orange).

We investigated whether the EEVD peptide competes for the binding site of a client protein, which could provide insights into key steps in the Sis1–Ssa1 interaction model. For this purpose, we used α-synuclein (α-syn), a well-known chaperone-interactor [18,34], as a client protein. We added α-syn to Sis1_1–352_ at a 2:1 stoichiometry and measured the resulting CSP (Figure 12). Significant CSPs were observed for residues in the GF region (S78, G91, G102, F106, and G126) and for residues in the β1/β2 region of the CTDI region (sites II and S189 at β1 and F201, K202, I203, and G204 at β2). Additional CSPs were detected at I217 and I219 in β3, K226 in the β3/β4 loop, and I231 in β4 (site I). CSPs were also noted at the end of β6 (I253 and E255), which are adjacent but not part of site I.

Many of the CTDI residues with significant CSPs are hydrophobic and play a role in stabilizing the β-sandwich fold of CTDI, which is formed by the β-hairpin β2/β3 and the β-sheet (β1, β4, β5, and β6). There are two hydrophobic pockets in the β-sandwich (client-binding pockets I and II): pocket II, located between β1 and β2, coincides with EEVD binding site II and is likely the primary client-binding site, as previously reported [35]. Pocket I, located at the opposite side between β3 and β4, is likely also an α-syn binding site. Notably, pocket I overlaps with EEVD binding site I, whereas pocket II, the primary α-syn binding site, shows the most pronounced CSP.

Importantly, a Sis1 triple mutant (Sis1 K199N/K202N/K214N), known to disrupt the α-syn binding region, does not bind the EEVD motif [36]. To test whether EEVD competes with α-syn for binding, we added a 10-fold excess of EEVD to the α-syn/Sis1_1–352_ sample. Competition was confirmed by the observation that residues showing significant CSP with α-syn exhibited even greater CSP after the addition of the EEVD peptide (Figure 12). Specifically, competition was detected at residue F106 in the GF region, as well as at residues S189, F201, K202, I203, and G204 in the hydrophobic pocket at site II of CTDI and at residues I217, I219, and I231 in the hydrophobic pocket at site I of CTDI. Competition was observed at residues I253 and E255 (Figure 12).

## 3. Conclusions

This study enhances our understanding of the structure and dynamics of the class B J domain protein (JDP) of *S. cerevisiae* (Sis1) in complex with the EEVD peptide of Ssa1.

This study successfully identified an EEVD site in the J-domain of Sis1. Upon binding of the EEVD peptide, numerous residues within the Sis1_1–81_ construct exhibited significant chemical shift variations in the ^1^H-^15^N-HSQC NMR spectra. In the bound state, the α2/α3 binding patch was found to be more open than in the free state. Additionally, in the bound conformation, α3 displayed a slight twist, likely adapting to accommodate the EEVD peptide. Molecular dynamics (MD) simulations play a crucial role in constructing the Sis1_1–81_:EEVD-peptide complex. By integrating data from chemical shift perturbations (CSPs) and paramagnetic relaxation enhancement (PRE), the simulations revealed that the peptide adopts an extended orientation between α2 and α3, thus positioning its N-terminus in close proximity to the HPD loop within the structural model of the Sis1_1–81_:EEVD peptide complex. Significantly, the simulations identified two salt bridges, K23-D8 and R27-E6, which were arranged in an i, i + 4 charged residue configuration. This arrangement is conserved in other DNAJB proteins, emphasizing the structural conservation across this protein family.

Solution studies of the interaction between the EEVD peptide and full-length Sis1 revealed that EEVD binds to multiple sites, including the J, GF, and CTDI domains. Additionally, the client protein α-synuclein induced significant chemical shift perturbations (CSPs) at three key locations: one in the intrinsically disordered region of the GF/GM and two in CTD1 (pockets I and II). These results strongly suggest that EEVD competes with the client protein for the GF and CTDI domains, particularly favoring site II (client-binding pocket II), as indicated by our results using α-syn. The interaction of EEVD with site I was previously observed in the crystal structure of its complex with DNAJB1 [32], but for Sis1, this interaction is reported here for the first time. The interaction of EEVD with site II in the CTDI domain of Sis1 was previously observed in the crystal structure of the Sis1 complex (constructed with only the CTDI/CTDII domains) and in solution through CSP, which also uses a construct containing only the CTDI/CTDII domains [35]. Notably, unlike Sis1, EEVD binds to the CTDII of *T. thermophilus* JDP (ttHsp40) [17].

## 4. Experimental Procedures

### 4.1. Sample Preparation

The expression and purification of Sis1_1–352_ (UNIPROT P25294) and Sis1_1–81_ were carried out following the procedures outlined in previous studies [6,19,21]. Additionally, the peptides GPTIEEVD and CGPTIEEVD, which correspond to the C-terminal motif of HSP70, were synthesized and purified by GenOne Biotechnologies (Rio de Janeiro, Brazil).

### 4.2. NMR Spectroscopy

The NMR data for Sis1_1–352_ were collected with a Bruker Avance III HD 950 MHz spectrometer equipped with ^13^C, ^15^N, ^1^H, and ^2^H TXI cryoprobe. Sis1_1–352_:EEVD-bound, Sis1_1–81_, and Sis1_1–81_:EEVD-bound samples were collected on a Bruker Avance III HD 900 MHz spectrometer equipped with an inverse-detection triple resonance z-gradient TXI probe. All the NMR samples contained 300 μM Sis1_1–352_ or 1 mM Sis1_1–81_ in 25 mM Tris-HCl (pH 7.5), 200 mM NaCl or 10% D_2_O at 303 K or 298 K, respectively. For further experimental details, see [21]. NMR data were processed and analyzed with nmrPipe [22] and CcpNmr Analysis [36], which are available on the NMRbox platform [37].

For backbone dynamics, the ^15^N backbone amide relaxation parameters (R_1_ and R_2_) were acquired on a Bruker 900 MHz instrument for ^15^N-Sis1_1–352_ at 303 K. TROSY-based ^15^N longitudinal (R_1_) relaxation rates were measured at 20, 300, and 600 ms, and transverse (R_2_) relaxation rates were measured at 8.65, 16.96, and 33.92 ms. Relaxation parameters were determined by fitting the T_1_ and T_2_ peak intensities to a single exponential decay. ^15^N R_1_ and R_2_ values were determined from the fit of the peak intensities via a monoexponential equation. The experimental errors of the relaxation parameters were evaluated based on the signal/noise ratio as described previously [38]. All relaxation experiments were acquired as pseudo3D spectra and converted to 2D datasets. NMR spectra were processed and analyzed with NMRPipe [39]. The overall rotational correlation time (τ_c_) of Sis1_1–81_ was estimated from the mean values of R_1_ and R_2_ measured for each domain as follows: $\tau c\approx \frac{1}{4\pi \upsilon_{N}}\sqrt{6\frac{R_{2}}{R_{1}}-7}$, where υ_N_ is the ^15^N resonance frequency (Hz) and R_1_ and R_2_ are the mean values of the ^15^N relaxation rates [40].

### 4.3. Chemical Shift Assignments of Sis1_1–81_:EEVD-Bound

For the backbone resonance assignments of Sis1_1–352_, data were acquired from the following two-dimensional (2D) and three-dimensional (3D) NMR experiments: all BEST-TROSY versions [40]: BT-HSQC, BT-^13^C-HSQC, BT-HNCACB, BT-HNCA, BT-HNCACO, BT-HNCO, BT-HNCOCA, BT-HNCOCACB, BT-N-NOESY, and BT-HNCANH. The assigned chemical shift values of backbone ^15^N, ^13^Cα, and ^13^C’ of Sis1_1–352_ were deposited in the BMRB (ID 51817) and used as inputs for the TALOS-N program [30,31] to predict secondary structures. The backbone and side chain assignments of Sis1_1–81_:EEVD-bound were previously documented and reported [21], and the data have been deposited in the Biological Magnetic Resonance Data Bank (BMRB; ID 51187).

### 4.4. Chemical Shift Mapping

To identify the interaction of Sis1_1–352_ or Sis1_1–81_ with the EEVD peptide, ^1^H-^15^N-TROSY spectra were collected for free ^15^N-labeled Sis1_1–352_ at 0.3 mM and upon the addition of GPTIEEVD- (from now on referred to as EEVD) (1:4 protein:peptide). For ^15^N-Sis1_1–81_ at 0.5 mM, ^1^H-^15^N HSQC spectra were collected free and upon the addition of EEVD (1:4 protein:peptide). For the interaction of Sis1_1–352_ with α-synuclein (α-syn) as a client protein, ^1^H-^15^N-TROSY spectra were collected for 0.3 mM free ^15^N-labeled Sis1_1–352_, and after the addition of 0.3 mM α-syn, a ~10-fold excess of EEVD, related to Sis1, was added to the same sample. Chemical shift perturbations (CSPs) were calculated for each backbone amide group as follows: $CSP=\sqrt{{\left({∆\delta }^{15N}/10\right)}^{2}+{\left({∆\delta }^{1H}\right)}^{2}}$, where Δδ^15N^ and Δδ^1H^ are the chemical shift differences between the free and bound states of Sis1_1–81_:EEVD. A CSP greater than 1 standard deviation from the mean was considered significant.

### 4.5. Structural Calculation of Sis1_1–81_:EEVD

NMR data for the structural calculation of Sis1_1–81_:EEVD were collected on a 1 mM ^13^C/^15^N-labeled Sis1_1–81_ in 25 mM Tris-HCl (pH 7.5), 200 mM NaCl and 10% D_2_O at 900 MHz. Distance restraints were derived from 3D ^15^N-NOESY-HSQC, an aliphatic 3D ^13^C-NOESY-HSQC and an aromatic ^13^C-NOESY-HSQC. Both the aliphatic and the aromatic and dihedral angle restraints were derived from the ^1^HN, ^15^N, ^13^Cα, ^1^Hα, ^13^Cβ, and C’ chemical shifts via TALOS-N [30,31]. The amino acid sequence, chemical shift lists, dihedral angle values, and three NOESY datasets were used as input files for structural determination.

Three-dimensional structures of Sis1_1–81_:EEVD-bound were calculated via the program Aria 2.3 [41,42] combined with CNS 1.2 [43] available on the NMRbox platform [37]. In the final step, we calculated 300 structures, selected 40 for water refinement, and reported the 20 lowest-energy structures from the water refinement as representative of the ensemble of Sis1_1–81_:EEVD conformations in solution. For quality validation, we used the protein structure validation software suite (PSVS) [44] (https://montelionelab.chem.rpi.edu/PSVS/PSVS/). The structures were visualized with PyMOL [45], and the atomic coordinates of Sis1_1–81_:EEVD were deposited in the *Protein Data Bank* under accession code 8EOD.

### 4.6. Site-Direct Spin-Labeling Experiments

A 20-fold excess of 4-maleimide-TEMPO (Sigma-Aldrich, St. Louis, MO, USA) dissolved in acetonitrile was added to the CGPTIEEVD peptide at a concentration of 1 mM in 25 mM Tris-HCl (pH 7.5) and 200 mM NaCl and incubated for two hours at room temperature. The final product was the TEMPO spin label attached to the reactive thiol functional group of Cys1. To remove unreacted and excess maleimide spin labels, the reaction mixture was injected into a C18 column, subjected to HPLC (Cytiva, Marlborough MA USA) in acetonitrile and water, and eluted with a 30–70% (water:acetonitrile 0.1% TFA) gradient.

### 4.7. Paramagnetic Relaxation Enhancement (PRE)

The direct interaction of Sis1_1–352_ and Sis1_1–81_ with the EEVD motif was investigated by analyzing the PRE with the TEMPO-labeled EEVD peptide. ^15^N-Sis1_1–81_ was titrated with TEMPO-labeled CGPTIEEVD peptide (paramagnetic sample), and ^1^H-^15^N HSQC spectra were recorded at 900 MHz. The diamagnetic samples were obtained after reduction with 3 mM ascorbic acid at room temperature for 4 h. PRE was obtained from the intensity ratio between the paramagnetic and diamagnetic spectra (${PRE=\alpha I}^{para}/I^{dia}$). We observed PRE effects greater than one because of the larger longitudinal relaxation time for the diamagnetic sample than for the paramagnetic sample and the short relaxation time used in the HSQC spectra (1.3 s). Therefore, we introduced a correction factor (α) to make the average <PRE> = 1 for the residues distant from the paramagnetic center.

To calibrate the PRE effect into semiquantitative distance ranges, we considered a linear change in the PRE effect as a function of the distance from each NH to the paramagnetic center (d) between 12 and 30 Å [29,30]. We considered d > 30 Å for PRE = 1 and d < 12 Å for PRE = 0. Distances between 12 and 30 Å were calculated (d_calc_), assuming a linear change proportional to the PRE. The distances were used semiquantitatively for the Haddock calculation: for PRE = 0 (peak disappears), 1.8 Å < d < 12 Å; for PRE between 0.8 and 0, d was in the range between 12 Å and d_calc_ + 3 Å.

### 4.8. Molecular Docking and Molecular Dynamics (MD) Simulations

Further details on the methodology and model construction are provided in the Supplementary Material: PART 2: Molecular Docking and Molecular Dynamic (MD) Simulations. Softwares: Haddock [24]; GROMACS (version 5.1.4) [46]; AMBER14-OL15 package, including the ff14sb protein force field [47], and the TIP3P water model [48]; tool *g_cluster* of the GROMACS [49], PyMOL [45].

## Figures and Tables

**Table 1:** Orientation and salt bridges from the CTDI:EEVD-peptide complexes at sites I and II.

Complex	Orientation	Salt-Bridges
Site I		
Lowest energy (Sis1, this work)	Parallel to β4	K234/D8, K230/E5
2nd Lowest energy (Sis1, this work)	Antiparallel to β4	K230/E6, K222/E5, K226/D8
DNAJB1 (crystal structure, 3AGY)	Antiparallel to β4 *	K217/E5 and K213/D8
Site II		
Lowest energy (Sis1, this work)	Parallel to β1 and anti-parallel to β2	K199/E6, K199/D8, K256/D8
2nd Lowest energy (Sis1, this work)	Parallel to β1 and anti-parallel to β2	K256/E5, K197/D8
DNAJB1 (crystal structure, 3AGY)	Antiparallel to β2 **	K181/E6, K184/E5, K182/D8
*Sis1* (crystal structure, 2B26)	Antiparallel to β2 **	K214/E6, K202/E6, K199/D8

* Electron density enables assertiveness of the orientation. ** Electron density does not enable assertiveness on the orientation.

## Data Availability

The original contributions presented in the study are included in the article/Supplementary Material, further inquiries can be directed to the corresponding authors.

## Supplementary Materials

The following supporting information can be downloaded at: https://www.mdpi.com/article/10.3390/molecules30010011/s1, Figure S1: Analysis of the 200 lowest energy structures calculated from Haddock via PRE and CSP restraints; Figure S2: EEVD peptide and interaction with the binding sites in the crystal structures of DNAJB1 and Sis1; Figure S3: Analysis of the parameters from 1.0 μs molecular dynamics simulations of the complex between CTDI sites I and II of JDPs and the EEVD peptide; Figure S4: Analysis of the stability between CTDI sites I and II of JDPs with the EEVD peptide from 1.0 μs molecular dynamics (MD) simulations; Figure S5: Electron density map of the crystal structures of JDPs bound to an EEVD peptide; Figure S6: Position and orientation of the EEVD peptide at CTDI sites I and II of JDPs; Figure S7: EEVD peptide interactions with site I of the JDPs CTDI; Figure S8: EEVD peptide interactions with site II of the JDPs CTDI; Figure S9: CTDI peptide binding site of Sis1; Figure S10: Alphafold2 prediction of Sis1; Table S1: NMR-derived restraints and structural statistics for the 20 best water-refined structures of the Sis1_1–81_:EEVD-bound conformation; Table S2: Results of molecular docking of the Sis1_1–81_:EEVD complex; Table S3: Hydrogen bonds between Sis_1–81_ and the EEVD peptide; Table S4: 15N longitudinal (R1) and transverse (R2) relaxation rates (R2/R1); Table S5: Semiquantitatively calibrated distance restraints; Table S6: Distance restraints semiquantitatively calibrated from the PRE data for Sis1_1–81_:EEVD used for molecular docking.

## Abbreviations

CSP: chemical shift perturbation; CTD, C-terminal domain; HSP, heat shock protein; HSP70, 70 kDa heat shock protein; IDR, intrinsically disordered region; JDP, J-domain protein; NBD, nucleotide-binding domain; R_1_, longitudinal relaxation rate; PRE, paramagnetic relaxation enhancement; R_2_, transverse relaxation rate; SBD, substrate binding domain; RMSD, root-mean-square deviation; Sis1, type B JDP from *Saccharomyces cerevisiae*; Ssa1, HSP70 from *Saccharomyces cerevisiae*.
