# Supplementary material for: NMR Studies on the Structure of Yeast Sis1 and the Dynamics of Its Interaction with Ssa1-EEVD
Source: Molecules. 2024 Dec 24;30(1):11. doi: 10.3390/molecules30010011 (PMC11722011; doi:10.3390/molecules30010011)
Supplement: Supplementary file 1 [file molecules-30-00011-s001.zip › molecules-3310973-supplementary.pdf]

Supplementary Material

NMR Studies on the Structure of Yeast Sis1 and the Dynamics of Its Interaction with Ssa1-EEVD

**Carolina O. Matos [1], Glaucia M. S. Pinheiro [1], Icaro P. Caruso [2], Gisele C. Amorim [3,4], Fabio C. L. Almeida [3,5,6,*]
and Carlos H. I. Ramos [1,5,*]**

1 Institute of Chemistry, University of Campinas UNICAMP, Campinas 13083-862, SP, Brazil;
carol.omatos@gmail.com (C.O.M.); glauciasquizato.pinheiro@hotmail.com (G.M.S.P.)
2 Multiuser Center for Biomolecular Innovation (CMIB), Department of Physics, São Paulo State University (UNESP), São Jose do Rio Preto 01049-010, SP, Brazil; icaro.caruso@unesp.br
3 National Center of Nuclear Magnetic Resonance (CNRMN), CENABIO, Federal University of Rio de Janeiro, Rio de Janeiro 21941-853, RJ, Brazil; gisele.amorim@caxias.ufrj.br
4 Multidisciplinary Center for Research in Biology (NUMPEX-Bio), Campus Duque de Caxias Federal University of Rio de Janeiro, Duque de Caxias 25240-005, RJ, Brazil
5 National Institute of Science and Technology for Bioimage and Structural Biology INBEB,
Rio de Janeiro 21941-902, RJ, Brazil
6 Institute of Medical Biochemistry, Federal University of Rio de Janeiro, Rio de Janeiro 21941-853, RJ, Brazil
* Correspondence: falmeida@bioqmed.ufrj.br (F.C.L.A.); cramos@unicamp.br (C.H.I.R.)

PART I: SUPPLEMENTARY TABLES

Table S1. NMR-derived restraints and structural statistics for the 20 best water-refined structures of the Sis1$_{1\text{-}81}$:EEVD-bound conformation.

Number of experimental restraints	
Total NOE distance restraints (ambiguous)	1206
Total NOE distance restraints (unambiguous)	2750
Total dihedral angle restraints	132
Ambiguous	
Short range ($\|I – j\| \leq 1$)	835
Medium range ($2 \leq \|I – j\| \leq 4$)	248
Long range ($\|I – j\| > 4$)	123
Unambiguous	
Short range ($\|I – j\| \leq 1$)	1821
Medium range ($2 \leq \|I – j\| \leq 4$)	560
Long range ($\|I – j\| > 4$)	369
Restraints violations	
distance constraints (>0.3 Å)	0
dihedral angle constraints (>5°)	0
RMSD from average structure (Å)	
Backbone (4-76)	0.8
Backbone, all residues	1.4
Heavy atoms (4-76)	1.2
Heavy atoms, all residues	1.7
Ramachandran plot of ordered residues – Procheck (%)	
Most favored regions	93.6
Allowed regions	6.4
Generously allowed	0
Disallowed	0
Ramachandran plot of ordered residues – Molprobity (%)	
Most favored regions	97.7

Allowed regions	2.2
Disallowed	0.1
CNS Energy Score (kcal/mol)	
E_{tot}	-3490 ± 88
E_{bound}	12.3 ± 0.6
E_{angle}	68 ± 4
E_{impr}	128 ±15
E_{dihed}	378 ± 4
E_{vdw}	-763 ± 10
E_{elec}	-3313 ± 88

Table S2: Results of molecular docking of the $Sis1_{1-81}$:EEVD complex

**Parameters**	**$Sis1_{1-81}$:EEVD complex**
HADDOCK score	-57.8 ± 6.5
Cluster size	7
RMSD	0.7 ± 0.5
van der Waals energy	-12 ± 11
Electrostatic energy	-242 ± 48
Desolvation energy	0 ± 3
Restraints violation energy	29 ± 14
Buried Surface Area	832 ± 32
Z score	-2.4

Table S3. Hydrogen bonds between $Sis_{1\text{-}81}$ and the EEVD peptide with more than 10% persistence throughout the 1 μs MD simulation are underlined. The residues and atoms of the EEVD peptide are underlined, whereas the residues and atoms of $Sis_{1\text{--}81}$ are not. Notably, some of the hydrogen bonds contribute to the formation of intermolecular salt bridges, thereby stabilizing the $Sis_{1\text{-}81}$:EEVD complex.

**Donor**	**Atom**	**Acceptor**	**Atom**	**Prevalence %**	**Salt Bridge**
GLU5	N	TYR26	OH	92.6	-
ASP8	N	ASN56	OD1	82.3	-
LYS23	NZ	GLU6	OE1	32.9	yes
HIS34	NE2	PRO2	O	32.1	-
LYS23	NZ	GLU6	OE2	30.2	yes
ASN56	ND2	ASP8	OC2	26.9	-
ASN56	ND2	ASP8	OC1	26.7	-
ARG27	NH2	GLU6	OE2	21.7	yes
ARG27	NH2	GLU6	OE1	21.4	yes

Table S4 ^{15}N longitudinal (R_1) and transverse (R_2) relaxation rates (R_2/R_1) and the apparent rotational diffusion (τ_c^{app}) of free and EEVD-bound $Sis1_{1\text{--}352}$

Domain (free or bound)	^{15}N-R_2/R_1	τ_c^{app}, s
J-domain (free)	22 ± 2	9.8 ± 1.0 ($x10^{-9}$)*
J-domain (bound)	20 ± 2	9.2 ± 1.0 ($x10^{-9}$)*
GF/GM region (free)	30 ± 13	11 ± 5 ($x10^{-9}$)
GF/GM region (bound)	22 ± 8	10 ± 4 ($x10^{-9}$)
CTD (free)	70 ± 8	18 ± 2 ($x10^{-9}$)
CTD (bound)	71 ± 8	18 ± 2 ($x10^{-9}$)

* τ_c^{app} of the bound domain is lower than that of a free domain at a significance level of $P < 0.05$

Table S5 | Semiquantitatively calibrated distance restraints from the PRE data for CTDI-$Sis1_{1\text{-}352}$:EEVD used for molecular docking

```
assign (segid "   A" and resid   187 and name   HN) (segid "   B" and resid    1 and name HN) 18.1 6.1 3 weight 1.000
assign (segid "   A" and resid   202 and name   HN) (segid "   B" and resid    1 and name HN) 26.8 14.8 3 weight 1.000
assign (segid "   A" and resid   206 and name   HN) (segid "   B" and resid    1 and name HN) 13.2 1.2 3 weight 1.000
assign (segid "   A" and resid   234 and name   HN) (segid "   B" and resid    1 and name HN) 26.9 14.9 3 weight 1.000
assign (segid "   A" and resid   240 and name   HN) (segid "   B" and resid    1 and name HN) 21.1 9.1 3 weight 1.000
assign (segid "   A" and resid   265 and name   HN) (segid "   B" and resid    1 and name HN) 22.5 10.5 3 weight 1.000
assign (segid "   A" and resid   286 and name   HN) (segid "   B" and resid    1 and name HN) 23.1 11.1 3 weight 1.000
assign (segid "   A" and resid   290 and name   HN) (segid "   B" and resid    1 and name HN) 25 13 3 weight 1.000
assign (segid "   A" and resid   331 and name   HN) (segid "   B" and resid    1 and name HN) 27.1 15.1 3 weight 1.000
assign (segid "   A" and resid   187 and name   HN) (segid "   B" and resid    1 and name N) 18.1 6.1 3 weight 1.000
assign (segid "   A" and resid   202 and name   HN) (segid "   B" and resid    1 and name N) 26.8 14.8 3 weight 1.000
assign (segid "   A" and resid   206 and name   HN) (segid "   B" and resid    1 and name N) 13.2 1.2 3 weight 1.000
assign (segid "   A" and resid   234 and name   HN) (segid "   B" and resid    1 and name N) 26.9 14.9 3 weight 1.000
assign (segid "   A" and resid   240 and name   HN) (segid "   B" and resid    1 and name N) 21.1 9.1 3 weight 1.000
assign (segid "   A" and resid   265 and name   HN) (segid "   B" and resid    1 and name N) 22.5 10.5 3 weight 1.000
assign (segid "   A" and resid   286 and name   HN) (segid "   B" and resid    1 and name N) 23.1 11.1 3 weight 1.000
assign (segid "   A" and resid   290 and name   HN) (segid "   B" and resid    1 and name N) 25 13 3 weight 1.000
assign (segid "   A" and resid   331 and name   HN) (segid "   B" and resid    1 and name N) 27.1 15.1 3 weight 1.000
assign (segid "   A" and resid   187 and name   HN) (segid "   B" and resid    1 and name CA) 18.1 6.1 3 weight 1.000
assign (segid "   A" and resid   202 and name   HN) (segid "   B" and resid    1 and name CA) 26.8 14.8 3 weight 1.000
assign (segid "   A" and resid   206 and name   HN) (segid "   B" and resid    1 and name CA) 13.2 1.2 3 weight 1.000
assign (segid "   A" and resid   234 and name   HN) (segid "   B" and resid    1 and name CA) 26.9 14.9 3 weight 1.000
assign (segid "   A" and resid   240 and name   HN) (segid "   B" and resid    1 and name CA) 21.1 9.1 3 weight 1.000
assign (segid "   A" and resid   265 and name   HN) (segid "   B" and resid    1 and name CA) 22.5 10.5 3 weight 1.000
assign (segid "   A" and resid   286 and name   HN) (segid "   B" and resid    1 and name CA) 23.1 11.1 3 weight 1.000
assign (segid "   A" and resid   290 and name   HN) (segid "   B" and resid    1 and name CA) 25 13 3 weight 1.000
assign (segid "   A" and resid   331 and name   HN) (segid "   B" and resid    1 and name CA) 27.1 15.1 3 weight 1.000
assign (segid "   A" and resid   187 and name   HN) (segid "   B" and resid    1 and name CB) 18.1 6.1 3 weight 1.000
assign (segid "   A" and resid   202 and name   HN) (segid "   B" and resid    1 and name CB) 26.8 14.8 3 weight 1.000
assign (segid "   A" and resid   206 and name   HN) (segid "   B" and resid    1 and name CB) 13.2 1.2 3 weight 1.000
assign (segid "   A" and resid   234 and name   HN) (segid "   B" and resid    1 and name CB) 26.9 14.9 3 weight 1.000
assign (segid "   A" and resid   240 and name   HN) (segid "   B" and resid    1 and name CB) 21.1 9.1 3 weight 1.000
assign (segid "   A" and resid   265 and name   HN) (segid "   B" and resid    1 and name CB) 22.5 10.5 3 weight 1.000
assign (segid "   A" and resid   286 and name   HN) (segid "   B" and resid    1 and name CB) 23.1 11.1 3 weight 1.000
assign (segid "   A" and resid   290 and name   HN) (segid "   B" and resid    1 and name CB) 25 13 3 weight 1.000
assign (segid "   A" and resid   331 and name   HN) (segid "   B" and resid    1 and name CB) 27.1 15.1 3 weight 1.000
assign (segid "   A" and resid   187 and name   HN) (segid "   B" and resid    1 and name C) 18.1 6.1 3 weight 1.000
assign (segid "   A" and resid   202 and name   HN) (segid "   B" and resid    1 and name C) 26.8 14.8 3 weight 1.000
assign (segid "   A" and resid   206 and name   HN) (segid "   B" and resid    1 and name C) 13.2 1.2 3 weight 1.000
assign (segid "   A" and resid   234 and name   HN) (segid "   B" and resid    1 and name C) 26.9 14.9 3 weight 1.000
assign (segid "   A" and resid   240 and name   HN) (segid "   B" and resid    1 and name C) 21.1 9.1 3 weight 1.000
assign (segid "   A" and resid   265 and name   HN) (segid "   B" and resid    1 and name C) 22.5 10.5 3 weight 1.000
assign (segid "   A" and resid   286 and name   HN) (segid "   B" and resid    1 and name C) 23.1 11.1 3 weight 1.000
assign (segid "   A" and resid   290 and name   HN) (segid "   B" and resid    1 and name C) 25 13 3 weight 1.000
assign (segid "   A" and resid   331 and name   HN) (segid "   B" and resid    1 and name C) 27.1 15.1 3 weight 1.000
assign (segid "   A" and resid   187 and name   HN) (segid "   B" and resid    1 and name HA) 18.1 6.1 3 weight 1.000
assign (segid "   A" and resid   202 and name   HN) (segid "   B" and resid    1 and name HA) 26.8 14.8 3 weight 1.000
assign (segid "   A" and resid   206 and name   HN) (segid "   B" and resid    1 and name HA) 13.2 1.2 3 weight 1.000
assign (segid "   A" and resid   234 and name   HN) (segid "   B" and resid    1 and name HA) 26.9 14.9 3 weight 1.000
assign (segid "   A" and resid   240 and name   HN) (segid "   B" and resid    1 and name HA) 21.1 9.1 3 weight 1.000
assign (segid "   A" and resid   265 and name   HN) (segid "   B" and resid    1 and name HA) 22.5 10.5 3 weight 1.000
assign (segid "   A" and resid   286 and name   HN) (segid "   B" and resid    1 and name HA) 23.1 11.1 3 weight 1.000
assign (segid "   A" and resid   290 and name   HN) (segid "   B" and resid    1 and name HA) 25 13 3 weight 1.000
assign (segid "   A" and resid   331 and name   HN) (segid "   B" and resid    1 and name HA) 27.1 15.1 3 weight 1.000
assign (segid "   A" and resid   187 and name   HN) (segid "   B" and resid    1 and name HB) 18.1 6.1 3 weight 1.000
assign (segid "   A" and resid   202 and name   HN) (segid "   B" and resid    1 and name HB) 26.8 14.8 3 weight 1.000
assign (segid "   A" and resid   206 and name   HN) (segid "   B" and resid    1 and name HB) 13.2 1.2 3 weight 1.000
assign (segid "   A" and resid   234 and name   HN) (segid "   B" and resid    1 and name HB) 26.9 14.9 3 weight 1.000
assign (segid "   A" and resid   240 and name   HN) (segid "   B" and resid    1 and name HB) 21.1 9.1 3 weight 1.000
assign (segid "   A" and resid   265 and name   HN) (segid "   B" and resid    1 and name HB) 22.5 10.5 3 weight 1.000
assign (segid "   A" and resid   286 and name   HN) (segid "   B" and resid    1 and name HB) 23.1 11.1 3 weight 1.000
assign (segid "   A" and resid   290 and name   HN) (segid "   B" and resid    1 and name HB) 25 13 3 weight 1.000
assign (segid "   A" and resid   331 and name   HN) (segid "   B" and resid    1 and name HB) 27.1 15.1 3 weight 1.000
```

Table S6 | Distance restraints semiquantitatively calibrated from the PRE data for $Sis1_{1-81}$:EEVD used for molecular docking

```
assign (segid "   A" and resid   10 and name   HN) (segid "   B" and resid   1 and name HN) 21 9 3 weight 1.000
assign (segid "   A" and resid   14 and name   HN) (segid "   B" and resid   1 and name HN) 26.7 14.7 3 weight 1.000
assign (segid "   A" and resid   24 and name   HN) (segid "   B" and resid   1 and name HN) 26.1 14.1 3 weight 1.000
assign (segid "   A" and resid   28 and name   HN) (segid "   B" and resid   1 and name HN) 17.4 5.4 3 weight 1.000
assign (segid "   A" and resid   30 and name   HN) (segid "   B" and resid   1 and name HN) 12 10.2 3 weight 1.000
assign (segid "   A" and resid   31 and name   HN) (segid "   B" and resid   1 and name HN) 16.2 4.2 3 weight 1.000
assign (segid "   A" and resid   32 and name   HN) (segid "   B" and resid   1 and name HN) 12.3 10.5 3 weight 1.000
assign (segid "   A" and resid   33 and name   HN) (segid "   B" and resid   1 and name HN) 14.3 12.5 3 weight 1.000
assign (segid "   A" and resid   34 and name   HN) (segid "   B" and resid   1 and name HN) 12.1 10.3 3 weight 1.000
assign (segid "   A" and resid   37 and name   HN) (segid "   B" and resid   1 and name HN) 31.3 19.3 3 weight 1.000
assign (segid "   A" and resid   40 and name   HN) (segid "   B" and resid   1 and name HN) 12.5 10.7 3 weight 1.000
assign (segid "   A" and resid   41 and name   HN) (segid "   B" and resid   1 and name HN) 17.8 5.8 3 weight 1.000
assign (segid "   A" and resid   43 and name   HN) (segid "   B" and resid   1 and name HN) 15.3 3.3 3 weight 1.000
assign (segid "   A" and resid   44 and name   HN) (segid "   B" and resid   1 and name HN) 16.2 4.2 3 weight 1.000
assign (segid "   A" and resid   45 and name   HN) (segid "   B" and resid   1 and name HN) 12 10.2 3 weight 1.000
assign (segid "   A" and resid   49 and name   HN) (segid "   B" and resid   1 and name HN) 12 10.2 3 weight 1.000
assign (segid "   A" and resid   54 and name   HN) (segid "   B" and resid   1 and name HN) 27.4 15.4 3 weight 1.000
assign (segid "   A" and resid   55 and name   HN) (segid "   B" and resid   1 and name HN) 29.5 17.5 3 weight 1.000
assign (segid "   A" and resid   10 and name   HN) (segid "   B" and resid   1 and name CA) 21 9 3 weight 1.000
assign (segid "   A" and resid   14 and name   HN) (segid "   B" and resid   1 and name CA) 26.7 14.7 3 weight 1.000
assign (segid "   A" and resid   24 and name   HN) (segid "   B" and resid   1 and name CA) 26.1 14.1 3 weight 1.000
assign (segid "   A" and resid   28 and name   HN) (segid "   B" and resid   1 and name CA) 17.4 5.4 3 weight 1.000
assign (segid "   A" and resid   30 and name   HN) (segid "   B" and resid   1 and name CA) 12 10.2 3 weight 1.000
assign (segid "   A" and resid   31 and name   HN) (segid "   B" and resid   1 and name CA) 16.2 4.2 3 weight 1.000
assign (segid "   A" and resid   32 and name   HN) (segid "   B" and resid   1 and name CA) 12.3 10.5 3 weight 1.000
assign (segid "   A" and resid   33 and name   HN) (segid "   B" and resid   1 and name CA) 14.3 12.5 3 weight 1.000
assign (segid "   A" and resid   34 and name   HN) (segid "   B" and resid   1 and name CA) 12.1 10.3 3 weight 1.000
assign (segid "   A" and resid   37 and name   HN) (segid "   B" and resid   1 and name CA) 31.3 19.3 3 weight 1.000
assign (segid "   A" and resid   40 and name   HN) (segid "   B" and resid   1 and name CA) 12.5 10.7 3 weight 1.000
assign (segid "   A" and resid   41 and name   HN) (segid "   B" and resid   1 and name CA) 17.8 5.8 3 weight 1.000
assign (segid "   A" and resid   43 and name   HN) (segid "   B" and resid   1 and name CA) 15.3 3.3 3 weight 1.000
assign (segid "   A" and resid   44 and name   HN) (segid "   B" and resid   1 and name CA) 16.2 4.2 3 weight 1.000
assign (segid "   A" and resid   45 and name   HN) (segid "   B" and resid   1 and name CA) 12 10.2 3 weight 1.000
assign (segid "   A" and resid   49 and name   HN) (segid "   B" and resid   1 and name CA) 12 10.2 3 weight 1.000
assign (segid "   A" and resid   54 and name   HN) (segid "   B" and resid   1 and name CA) 27.4 15.4 3 weight 1.000
assign (segid "   A" and resid   55 and name   HN) (segid "   B" and resid   1 and name CA) 29.5 17.5 3 weight 1.000
assign (segid "   A" and resid   10 and name   HN) (segid "   B" and resid   1 and name HA) 21 9 3 weight 1.000
assign (segid "   A" and resid   14 and name   HN) (segid "   B" and resid   1 and name HA) 26.7 14.7 3 weight 1.000
assign (segid "   A" and resid   24 and name   HN) (segid "   B" and resid   1 and name HA) 26.1 14.1 3 weight 1.000
assign (segid "   A" and resid   28 and name   HN) (segid "   B" and resid   1 and name HA) 17.4 5.4 3 weight 1.000
assign (segid "   A" and resid   30 and name   HN) (segid "   B" and resid   1 and name HA) 12 10.2 3 weight 1.000
assign (segid "   A" and resid   31 and name   HN) (segid "   B" and resid   1 and name HA) 16.2 4.2 3 weight 1.000
assign (segid "   A" and resid   32 and name   HN) (segid "   B" and resid   1 and name HA) 12.3 10.5 3 weight 1.000
assign (segid "   A" and resid   33 and name   HN) (segid "   B" and resid   1 and name HA) 14.3 12.5 3 weight 1.000
assign (segid "   A" and resid   34 and name   HN) (segid "   B" and resid   1 and name HA) 12.1 10.3 3 weight 1.000
assign (segid "   A" and resid   37 and name   HN) (segid "   B" and resid   1 and name HA) 31.3 19.3 3 weight 1.000
assign (segid "   A" and resid   40 and name   HN) (segid "   B" and resid   1 and name HA) 12.5 10.7 3 weight 1.000
assign (segid "   A" and resid   41 and name   HN) (segid "   B" and resid   1 and name HA) 17.8 5.8 3 weight 1.000
assign (segid "   A" and resid   43 and name   HN) (segid "   B" and resid   1 and name HA) 15.3 3.3 3 weight 1.000
assign (segid "   A" and resid   44 and name   HN) (segid "   B" and resid   1 and name HA) 16.2 4.2 3 weight 1.000
assign (segid "   A" and resid   45 and name   HN) (segid "   B" and resid   1 and name HA) 12 10.2 3 weight 1.000
assign (segid "   A" and resid   49 and name   HN) (segid "   B" and resid   1 and name HA) 12 10.2 3 weight 1.000
assign (segid "   A" and resid   54 and name   HN) (segid "   B" and resid   1 and name HA) 27.4 15.4 3 weight 1.000
assign (segid "   A" and resid   55 and name   HN) (segid "   B" and resid   1 and name HA) 29.5 17.5 3 weight 1.000
assign (segid "   A" and resid   10 and name   HN) (segid "   B" and resid   1 and name N) 21 9 3 weight 1.000
assign (segid "   A" and resid   14 and name   HN) (segid "   B" and resid   1 and name N) 26.7 14.7 3 weight 1.000
assign (segid "   A" and resid   24 and name   HN) (segid "   B" and resid   1 and name N) 26.1 14.1 3 weight 1.000
assign (segid "   A" and resid   28 and name   HN) (segid "   B" and resid   1 and name N) 17.4 5.4 3 weight 1.000
assign (segid "   A" and resid   30 and name   HN) (segid "   B" and resid   1 and name N) 12 10.2 3 weight 1.000
assign (segid "   A" and resid   31 and name   HN) (segid "   B" and resid   1 and name N) 16.2 4.2 3 weight 1.000
assign (segid "   A" and resid   32 and name   HN) (segid "   B" and resid   1 and name N) 12.3 10.5 3 weight 1.000
```

```
assign (segid "   A" and resid  33 and name   HN) (segid "   B" and resid     1 and name N) 14.3 12.5 3 weight 1.000
assign (segid "   A" and resid  34 and name   HN) (segid "   B" and resid     1 and name N) 12.1 10.3 3 weight 1.000
assign (segid "   A" and resid  37 and name   HN) (segid "   B" and resid     1 and name N) 31.3 19.3 3 weight 1.000
assign (segid "   A" and resid  40 and name   HN) (segid "   B" and resid     1 and name N) 12.5 10.7 3 weight 1.000
assign (segid "   A" and resid  41 and name   HN) (segid "   B" and resid     1 and name N) 17.8 5.8 3 weight 1.000
assign (segid "   A" and resid  43 and name   HN) (segid "   B" and resid     1 and name N) 15.3 3.3 3 weight 1.000
assign (segid "   A" and resid  44 and name   HN) (segid "   B" and resid     1 and name N) 16.2 4.2 3 weight 1.000
assign (segid "   A" and resid  45 and name   HN) (segid "   B" and resid     1 and name N) 12 10.2 3 weight 1.000
assign (segid "   A" and resid  49 and name   HN) (segid "   B" and resid     1 and name N) 12 10.2 3 weight 1.000
assign (segid "   A" and resid  54 and name   HN) (segid "   B" and resid     1 and name N) 27.4 15.4 3 weight 1.000
assign (segid "   A" and resid  55 and name   HN) (segid "   B" and resid     1 and name N) 29.5 17.5 3 weight 1.000
assign (segid "   A" and resid  10 and name   HN) (segid "   B" and resid     1 and name C) 21 9 3 weight 1.000
assign (segid "   A" and resid  14 and name   HN) (segid "   B" and resid     1 and name C) 26.7 14.7 3 weight 1.000
assign (segid "   A" and resid  24 and name   HN) (segid "   B" and resid     1 and name C) 26.1 14.1 3 weight 1.000
assign (segid "   A" and resid  28 and name   HN) (segid "   B" and resid     1 and name C) 17.4 5.4 3 weight 1.000
assign (segid "   A" and resid  30 and name   HN) (segid "   B" and resid     1 and name C) 12 10.2 3 weight 1.000
assign (segid "   A" and resid  31 and name   HN) (segid "   B" and resid     1 and name C) 16.2 4.2 3 weight 1.000
assign (segid "   A" and resid  32 and name   HN) (segid "   B" and resid     1 and name C) 12.3 10.5 3 weight 1.000
assign (segid "   A" and resid  33 and name   HN) (segid "   B" and resid     1 and name C) 14.3 12.5 3 weight 1.000
assign (segid "   A" and resid  34 and name   HN) (segid "   B" and resid     1 and name C) 12.1 10.3 3 weight 1.000
assign (segid "   A" and resid  37 and name   HN) (segid "   B" and resid     1 and name C) 31.3 19.3 3 weight 1.000
assign (segid "   A" and resid  40 and name   HN) (segid "   B" and resid     1 and name C) 12.5 10.7 3 weight 1.000
assign (segid "   A" and resid  41 and name   HN) (segid "   B" and resid     1 and name C) 17.8 5.8 3 weight 1.000
assign (segid "   A" and resid  43 and name   HN) (segid "   B" and resid     1 and name C) 15.3 3.3 3 weight 1.000
assign (segid "   A" and resid  44 and name   HN) (segid "   B" and resid     1 and name C) 16.2 4.2 3 weight 1.000
assign (segid "   A" and resid  45 and name   HN) (segid "   B" and resid     1 and name C) 12 10.2 3 weight 1.000
assign (segid "   A" and resid  49 and name   HN) (segid "   B" and resid     1 and name C) 12 10.2 3 weight 1.000
assign (segid "   A" and resid  54 and name   HN) (segid "   B" and resid     1 and name C) 27.4 15.4 3 weight 1.000
assign (segid "   A" and resid  55 and name   HN) (segid "   B" and resid     1 and name C) 29.5 17.5 3 weight 1.000
assign (segid "   A" and resid  10 and name   HN) (segid "   B" and resid     1 and name HB) 21 9 3 weight 1.000
assign (segid "   A" and resid  14 and name   HN) (segid "   B" and resid     1 and name HB) 26.7 14.7 3 weight 1.000
assign (segid "   A" and resid  24 and name   HN) (segid "   B" and resid     1 and name HB) 26.1 14.1 3 weight 1.000
assign (segid "   A" and resid  28 and name   HN) (segid "   B" and resid     1 and name HB) 17.4 5.4 3 weight 1.000
assign (segid "   A" and resid  30 and name   HN) (segid "   B" and resid     1 and name HB) 12 10.2 3 weight 1.000
assign (segid "   A" and resid  31 and name   HN) (segid "   B" and resid     1 and name HB) 16.2 4.2 3 weight 1.000
assign (segid "   A" and resid  32 and name   HN) (segid "   B" and resid     1 and name HB) 12.3 10.5 3 weight 1.000
assign (segid "   A" and resid  33 and name   HN) (segid "   B" and resid     1 and name HB) 14.3 12.5 3 weight 1.000
assign (segid "   A" and resid  34 and name   HN) (segid "   B" and resid     1 and name HB) 12.1 10.3 3 weight 1.000
assign (segid "   A" and resid  37 and name   HN) (segid "   B" and resid     1 and name HB) 31.3 19.3 3 weight 1.000
assign (segid "   A" and resid  40 and name   HN) (segid "   B" and resid     1 and name HB) 12.5 10.7 3 weight 1.000
assign (segid "   A" and resid  41 and name   HN) (segid "   B" and resid     1 and name HB) 17.8 5.8 3 weight 1.000
assign (segid "   A" and resid  43 and name   HN) (segid "   B" and resid     1 and name HB) 15.3 3.3 3 weight 1.000
assign (segid "   A" and resid  44 and name   HN) (segid "   B" and resid     1 and name HB) 16.2 4.2 3 weight 1.000
assign (segid "   A" and resid  45 and name   HN) (segid "   B" and resid     1 and name HB) 12 10.2 3 weight 1.000
assign (segid "   A" and resid  49 and name   HN) (segid "   B" and resid     1 and name HB) 12 10.2 3 weight 1.000
assign (segid "   A" and resid  54 and name   HN) (segid "   B" and resid     1 and name HB) 27.4 15.4 3 weight 1.000
assign (segid "   A" and resid  55 and name   HN) (segid "   B" and resid     1 and name HB) 29.5 17.5 3 weight 1.000
assign (segid "   A" and resid  10 and name   HN) (segid "   B" and resid     1 and name CB) 21 9 3 weight 1.000
assign (segid "   A" and resid  14 and name   HN) (segid "   B" and resid     1 and name CB) 26.7 14.7 3 weight 1.000
assign (segid "   A" and resid  24 and name   HN) (segid "   B" and resid     1 and name CB) 26.1 14.1 3 weight 1.000
assign (segid "   A" and resid  28 and name   HN) (segid "   B" and resid     1 and name CB) 17.4 5.4 3 weight 1.000
assign (segid "   A" and resid  30 and name   HN) (segid "   B" and resid     1 and name CB) 12 10.2 3 weight 1.000
assign (segid "   A" and resid  31 and name   HN) (segid "   B" and resid     1 and name CB) 16.2 4.2 3 weight 1.000
assign (segid "   A" and resid  32 and name   HN) (segid "   B" and resid     1 and name CB) 12.3 10.5 3 weight 1.000
assign (segid "   A" and resid  33 and name   HN) (segid "   B" and resid     1 and name CB) 14.3 12.5 3 weight 1.000
assign (segid "   A" and resid  34 and name   HN) (segid "   B" and resid     1 and name CB) 12.1 10.3 3 weight 1.000
assign (segid "   A" and resid  37 and name   HN) (segid "   B" and resid     1 and name CB) 31.3 19.3 3 weight 1.000
assign (segid "   A" and resid  40 and name   HN) (segid "   B" and resid     1 and name CB) 12.5 10.7 3 weight 1.000
assign (segid "   A" and resid  41 and name   HN) (segid "   B" and resid     1 and name CB) 17.8 5.8 3 weight 1.000
assign (segid "   A" and resid  43 and name   HN) (segid "   B" and resid     1 and name CB) 15.3 3.3 3 weight 1.000
assign (segid "   A" and resid  44 and name   HN) (segid "   B" and resid     1 and name CB) 16.2 4.2 3 weight 1.000
assign (segid "   A" and resid  45 and name   HN) (segid "   B" and resid     1 and name CB) 12 10.2 3 weight 1.000
assign (segid "   A" and resid  49 and name   HN) (segid "   B" and resid     1 and name CB) 12 10.2 3 weight 1.000
assign (segid "   A" and resid  54 and name   HN) (segid "   B" and resid     1 and name CB) 27.4 15.4 3 weight 1.000
assign (segid "   A" and resid  55 and name   HN) (segid "   B" and resid     1 and name CB) 29.5 17.5 3 weight 1.000
```

PART 2: **Molecular docking and molecular dynamics (MD) simulations.**

2.1 Methodology.

The software Haddock was used to dock the EEVD peptide to the EEVD-bound conformation of $Sis1_{1\text{-}81}$ (PDB 8EOD) via residues with CSPs as active residues and distance restraints semiquantitatively calibrated from the PRE data. The passive residues were automatically assigned as those surrounding the active residues. The HADDOCK (version 2.4) server (https://wenmr.science.uu.nl/haddock2.4/) was used for the modeling of $Sis1_{1\text{-}81}$:EEVD. In total, 2000 complex structures of rigid-body docking were calculated via the standard HADDOCK protocol with an optimized potential for liquid simulation (OPLSX) parameters. The final 200 lowest-energy structures were selected for subsequent explicit solvent (water) and semiflexible simulated annealing refinement to optimize the side chain constants. The final structures were clustered using the fraction of common contacts (FCC) with a cutoff of 0.6.

The software Haddock was used to dock the EEVD peptide to the CTDI (residues 180--257) of $Sis1_{1\text{--}352}$ using the residues with CSP as the active residues and distance restraints semiquantitatively calibrated from the PRE data (Tables S2 and S3). The passive residues were automatically assigned as those surrounding the active residues. Residues with low-order parameters ($S^2 < 0.6$) were set as fully flexible. For $Sis1_{1\text{-}352}$, we have NMR-based interaction information with many regions but created docking models for CTDI only. It was unfeasible to create an interaction model of one EEVD peptide that was compatible with all the experimental data. The HADDOCK (version 2.4) server (https://wenmr.science.uu.nl/haddock2.4/) was used for modeling the CTDI:EEVD. The protein structural coordinates of the CTDI used as input were obtained from an AlphaFold prediction, which is almost identical to the Protein Data Bank (PDB) under access code 2B26. In total, 2000 complex structures of rigid-body docking were calculated via the standard HADDOCK protocol with an optimized potential for liquid simulation (OPLSX) parameters. The final 200 lowest-energy structures were selected for subsequent explicit solvent (water) and semiflexible simulated annealing refinement to optimize the side chain constants. The VolMap tool of visual molecular dynamics (VMD) software was used for the construction of the atomistic probability map of CTDI-Sis1:EEVD. An ensemble of the 100 lowest-energy structures calculated from Haddock was used. The 100 structures were clustered into two groups. Cluster I included all the poses binding to Site I, and Cluster II included all the poses binding to Site II.

Molecular dynamics (MD) calculations for docking models of CTDIs at sites I and II and the 2 lowest energy structures of the highest representative cluster of the $Sis1_{1\text{-}81}$:EEVD complex were performed via GROMACS (version 5.1.4). The molecular systems were modeled with the corrected AMBER14-OL15 package, including the ff14sb protein force field, as well as the TIP3P water model. The structural models of the complexes (from molecular docking) were placed in

the center of a cubic box solvated by a solution of 200 mM NaCl in water. Periodic boundary conditions were used, and all simulations were performed in an NPT ensemble, keeping the system at 25 °C and 1.0 bar using a Nose–Hoover thermostat (τ_T = 2 ps) and Parrinello-Rahman barostat (τ_P = 2 ps and compressibility = $4.5\times10^{-5}\cdot bar^{-1}$). A cutoff of 12 Å for both the Lennard–Jones and Coulomb potentials was used. The long-range electrostatic interactions were calculated via the particle mesh Ewald (PME) algorithm. A conjugate gradient minimization algorithm was used to relax the superposition of atoms generated in the box construction process. Energy minimizations were carried out with the steepest descent integrator and conjugate gradient algorithm, using 1,000 $kJ\cdot mol^{-1}\cdot nm^{-1}$ as the maximum force criterion. Five hundred thousand steps of molecular dynamics were performed for NVT and NPT equilibration, applying force constants of 1,000 $kJ\cdot mol^{-1}\cdot nm^{-2}$ to all heavy atoms of the complex. At the end of preparation, a 100 ns MD pulling simulation of the molecular system was carried out using a spring constant of 1,000 $kJ\cdot mol^{-1}\cdot nm^{-2}$ between the protein and the peptide. Next, a 1 μs MD simulation was performed for data acquisition. Following the dynamic process, the trajectories of the complex were first concatenated and analyzed according to the RMSD for the backbone atoms of the protein and peptide; many contacts for distances lower than 0.6 nm between pairs of atoms of the CTDI-Sis1/Sis$1_{1\text{-}81}$ and EEVD peptides; and several protein–peptide hydrogen bonds with a cutoff distance (heavy atoms) of 3.5 Å and a maximum angle of 30°. The percentages of protein–peptide hydrogen bond persistence were obtained from *plot_hbmap_generic.pl* script. The number of protein–peptide hydrogen bonds with a persistence greater than 10% was considered. The tool *g_cluster* of the GROMACS[57] package was used to perform cluster analysis in the 1.0 μs MD trajectory of the complex using a cutoff of 3 Å. The structure of the first cluster was used as a representative structure of the complex. The structural representation of the constructed model was displayed via PyMOL.

2.2. CTDI-EEVD

Initially, the docking targeted the dimeric CTDI (180-257) using only the PRE and CSP data specific to this region. We observed that the EEVD peptide binds to the CTDI at two primary interaction sites, named sites I and II, which are located on opposite faces of the domain (Fig. S1). While the interaction space at the CTDI (sites I and II) was well defined, the orientation and position of the EEVD peptide relative to these sites were not well defined. Our analysis strategy focused on describing the interaction space within the CTDI, on the basis of the numerous observed poses of the EEVD relative to this domain (Fig. S1c). To achieve this, we selected the 100 conformers with the lowest

binding energies, which ranged from -399.8 to -174.0 kcal/mol (Fig. S1a). Similar results would have been obtained if we had chosen the 100 structures with the lowest total energies because of the linear correlation observed between the complex energy and binding energy (Fig. S1b). By using the 100 lowest binding energy structures, we ensured that all selected structures had negative complexes and binding energies (Fig. S1b), representing well-behaved complex models in terms of geometry. Next, we separated the 100 structures into two clusters: cluster I, containing all the poses that bind to site I (73 structures), and cluster II, containing the poses that bind to site II (27 structures) (Fig. S1c). Using *VolMap 1.9.3*, a structural tool of VMD software, we calculated the atomistic (mass) probability density map for both clusters. Figs. S1d and S1e show the lowest binding energy poses, which superpose well with the calculated density maps.

The position of the density maps matches the experimental PRE and CSP in the CTDI, as measured for $Sis1_{1\text{--}352}$ in the presence of the EEVD peptide (Fig S2). For site I (Fig S2a), the density map also coincides exactly with the position of an HSP70-EEVD peptide complexed with human DNAJB1, and one of the poses even forms an antiparallel β-strand with the CTDI β-strand 4 (β4), the same configuration of the EEVD complex at site I of human DNAB1. For site II, the density map is between β1 and β2, while both crystal structures describe the interaction of the EEVD peptide with either DNAB1 (3AGY; Fig S2b) or Sis1 (2B26; Fig S2c), which interact with β2, forming an antiparallel β-strand.

To determine the stability of the docking presented here, we run 100 ns molecular dynamics (MD) simulations of the 2 lowest energy docking poses for each site (I and II). As a control, we also run MD simulations starting from the crystallographic structure of EEVD complexed with DNAJB1 and Sis1 at sites I and II (Figs. S3 and S4). The docked structures were stable during the MD simulations.

In the crystal structure of DNAJB1 complexed with the EEVD peptide (PDB 3AGY), at site II, the same observations are valid, with low electron density for the EEVD-peptide backbone and no electron density for the side chains (Fig. S5c and S5d). For DNAJB1 at site I, the electron density of the backbone is well defined, and sidechains P2, I4, E5, and E6 have reasonably well-defined electron densities, indicating the relative orientation of the EEVD peptide and β4 as antiparallel β strands (Fig. S5c and S5e).

To obtain further evidence regarding the presence of two binding sites on the CTDI of Sis1, we analyzed the electron density maps of all available crystal structures of JDPs describing the interaction of the EEVD peptide and CTDI. In the crystal structure of Sis1$_{171–352}$ complexed with the EEVD peptide (PDB 2B26), the backbone of the EEVD peptide bound to site II is well defined. However, none of the side chains display clear electron density, making it challenging to determine the orientation of the peptide relative to β2 (Fig. S6a and b). Notably, in this case, the EEVD peptide is not involved in any crystal contact. Similarly, in the crystal structure of DNAJB1 complexed with the EEVD peptide (PDB 3AGY) at site II, the same observations apply: the backbone of the EEVD peptide has low electron density, and there is no discernible density for the side chains (Fig. S6c and d). For DNAJB1 at site I, the electron density for the backbone is well defined, and the side chains of residues P2, I4, E5, and E6 have reasonably well-defined electron densities. This allows for more accurate determination of the relative orientation of the EEVD peptide and β4, indicating antiparallel β-strand alignment (Fig. S6c and e).

The analysis of the lowest energy docking models generated in this work for site I from Sis1, in which the atomic probability map coincides with the electron density map of the crystal structures, allows for two possible orientations of the peptide relative to β4 (Fig. S6). The EEVD peptide with the lowest energy (Fig. S6a) has a parallel orientation relative to that of CTDI-β4, whereas the 2nd lowest energy structure has an antiparallel orientation to this domain (Fig. 12B), similar to the EEVD peptide in DNAJB1 at site I (Fig. S6c). Notably, both orientations are stabilized by a considerable number of salt bridges and hydrophobic and hydrogen bonding interactions (Fig. S7 and Table 1). The relative orientation to β2 of the lowest energy complexes is the same as that depicted in the crystal structures (antiparallel to β2, Fig. S6d-g). The lowest-energy structures are stabilized by a considerable number of salt bridges (Table 1, Fig. S8), hydrogen bonds, and hydrophobic interactions.

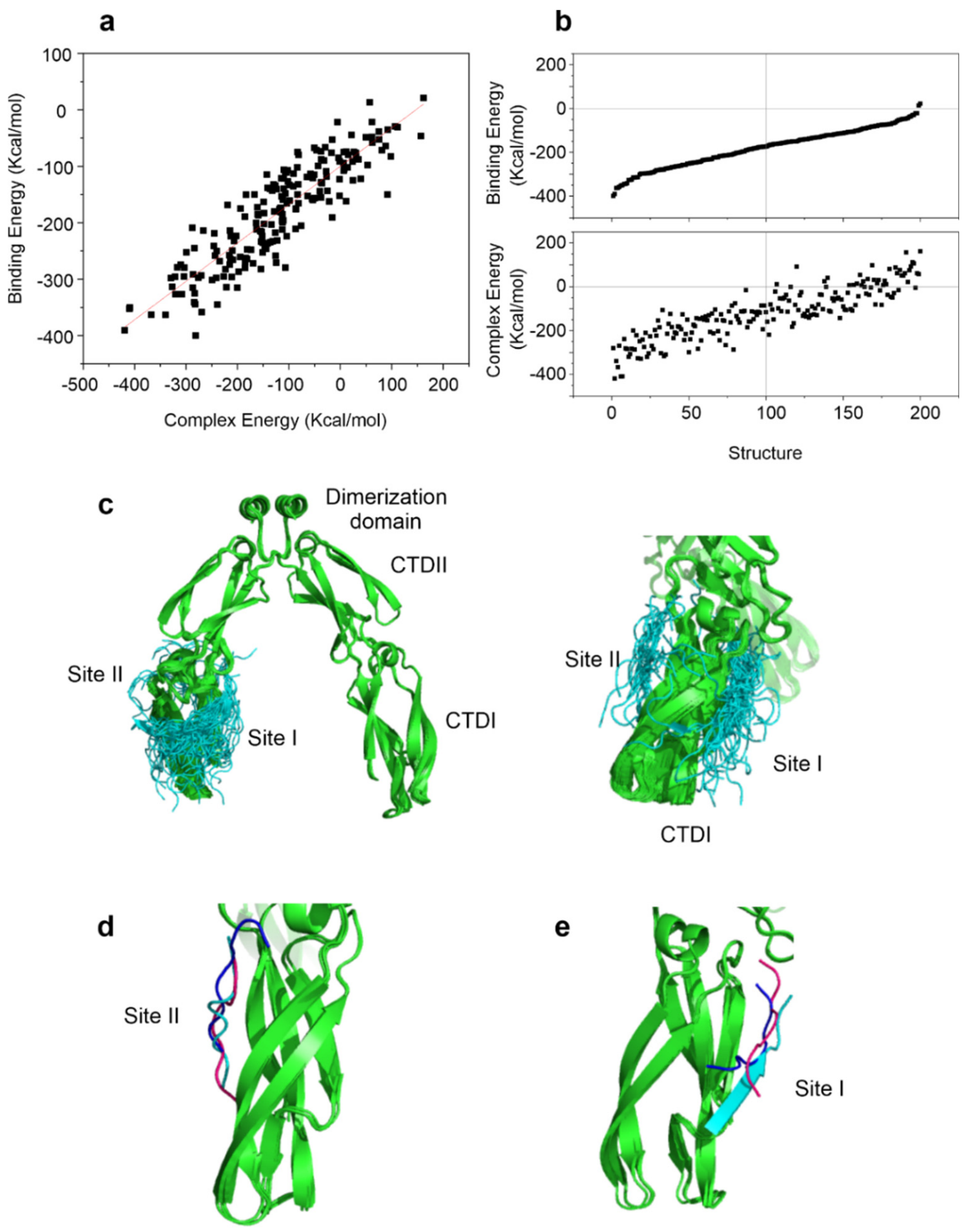

Figure S1 Analysis of the 200 lowest energy structures calculated from Haddock via PRE and CSP restraints. A Binding energy as a function of complex energy. **B** Binding energy and energy of the CTDI:EEVD complex as a function of the 200 docked structures. Note that the 100 lowest energy structures had both negative binding and CTDI:EEVD complex energies. We chose to construct the mass density plot from the clusters at sites I and II of these 100 energy poses. **c** Cartoon representation of the superposition of the 100 lowest binding energies bound to sites I and II of CTDIs. The EEVD peptide is in cyan, and Sis1 is in green. **D** Cartoon representation of the superposition of the 3 lowest binding energy poses of the EEVD peptide (cyan, dark blue and pink) bound to site II. **E** Cartoon representation of the superposition of the 3 lowest binding energy poses of the EEVD peptide (cyan, dark blue and pink) bound to site I.

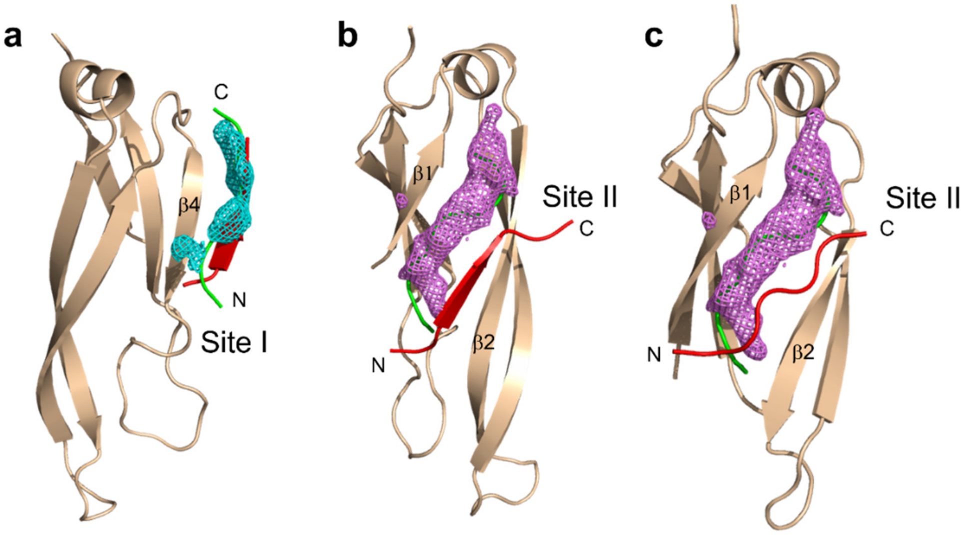

Fig. S2 | EEVD peptide and interaction with the binding sites in the crystal structures of DNAJB1 and Sis1. Cartoon representation of the CTDI (wheat color) from **A** DNAJB1 site I according to its crystal structure (PDB 3AGY). The mass density map is in cyan (meshes). The EEVD peptide from the crystal structure is in red, and the lowest energy poses are in green. **B** DNAJB1 site II according to its crystal structure (PDB 3AGY). The EEVD peptide from the crystal structure is in red. The mass density map of the EEVD peptide is shown in magenta (meshes), and the lowest structure is shown in green. **C** Sis1-site II (PDB 2B26) DNAJB1-site II according to its crystal structure (PDB 3AGY). The EEVD peptide from the crystal structure is in red. The mass density map of the EEVD peptide is shown in magenta (meshes), and the lowest structure is shown in green.

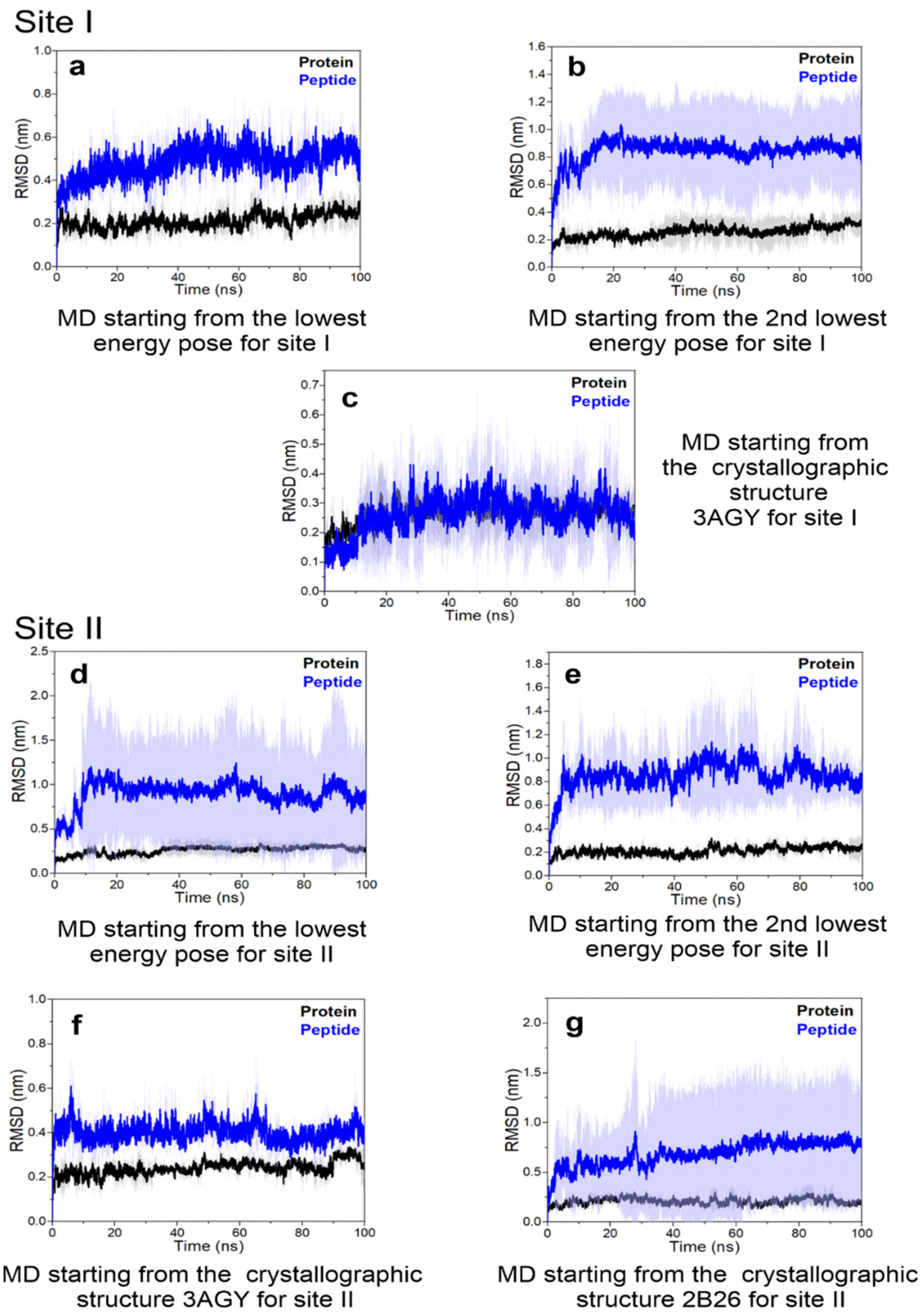

Fig. S3 | Analysis of the parameters from 1.0 µs molecular dynamics simulations of the complex between CTDI sites I and II of JDPs and the EEVD peptide. a, d Values of the root mean square deviations (RMSDs) of the backbone atoms of Sis1-CTDI from the lowest energy pose for site I and site II (black), respectively, and the EEVD peptide (blue). **b, e** RMSDs of the backbone atoms of Sis1-CTDI from the second lowest energy pose for site I and site II (black), respectively, and the EEVD peptide (blue). **c, f** Values of RMSDs of the backbone atoms of DNAJB1-CTDI from the crystallographic structure (PDB 3AGY) for site I and site II (black), respectively, and the EEVD peptide (blue). **g** RMSDs of the backbone atoms of Sis1-CTDI from the crystallographic structure (PDB 2B26) for site II (black) and the EEVD peptide (blue).

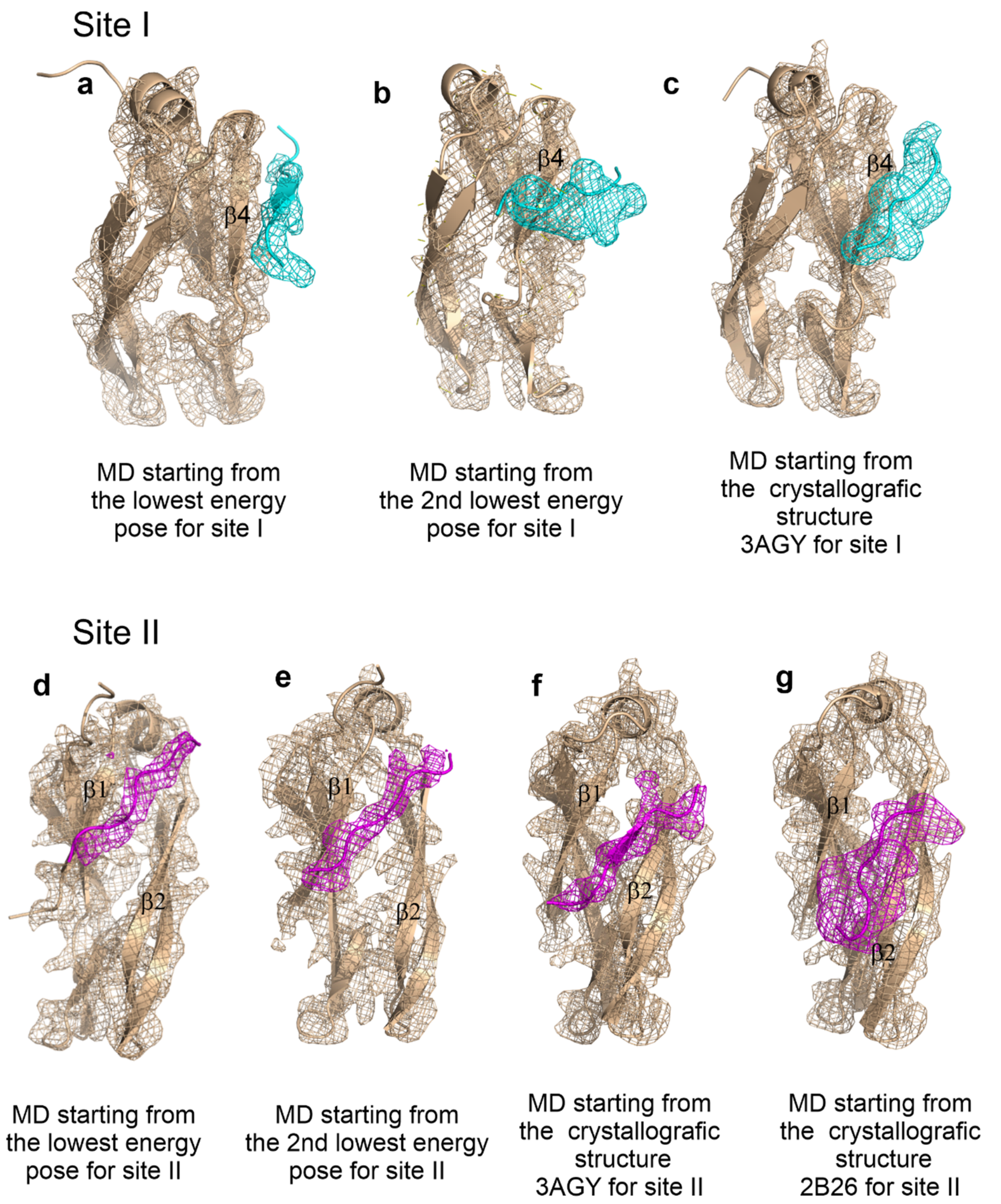

Fig. S4 | Analysis of the stability between CTDI sites I and II of JDPs with the EEVD peptide from 1.0 µs molecular dynamics (MD) simulations. Cartoon representation of the CTDI and the mass density map (wheat color) from **a** the lowest energy pose for site I; **b** the second lowest energy pose for site I; **c** DNAJB1-site I according to its crystal structure (PDB 3AGY). The EEVD peptide and its mass density map are in cyan (meshes); **d** the lowest energy pose for site II **e** the second lowest energy pose for site II; **f** DNAJB1-site II according to its crystal structure (PDB 3AGY); and **g** Sis1-site II according to its crystal structure (PDB 2B26). The EEVD peptide and its mass density map are shown in magenta (meshes).

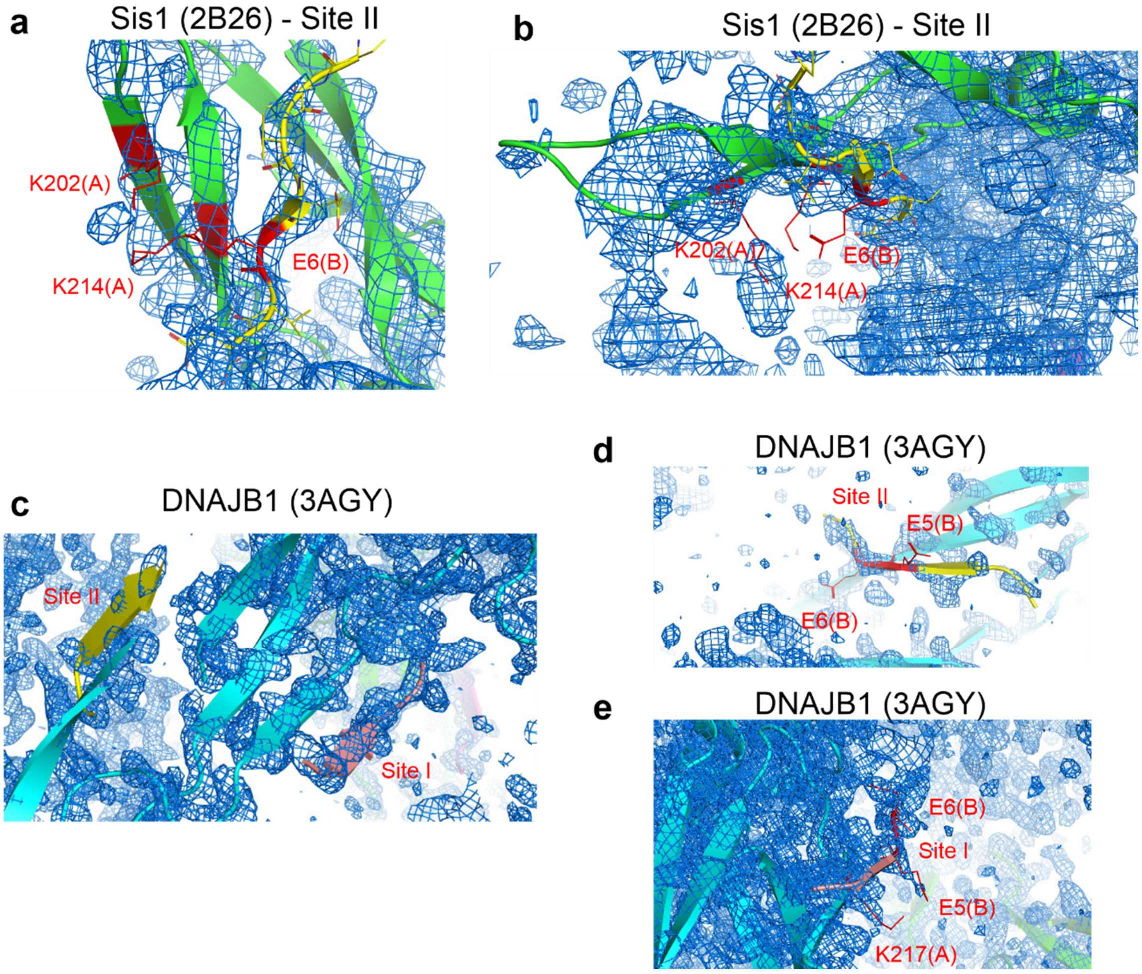

Fig. S5 | Electron density map of the crystal structures of JDPs bound to an EEVD peptide. a and **b** Cartoon representation of the CTDI of Sis1 (PDB 2B26) (green) with the EEVD peptide (yellow) bound at site II. The electron density map is in blue. Note that the EEVD backbone has a well-defined electron density, but side-chain information is lacking. **c-e** Cartoon representation of the CTDI of DNAJB1 (PDB 3AGY) (cyan) with the EEVD peptide in yellow when bound to site II and in pink when bound to site I. The electron density map is blue and lacks resolution at site II (**d**) but not at site I (**e**), which is well defined for residues P2, I4, E5 and E6.

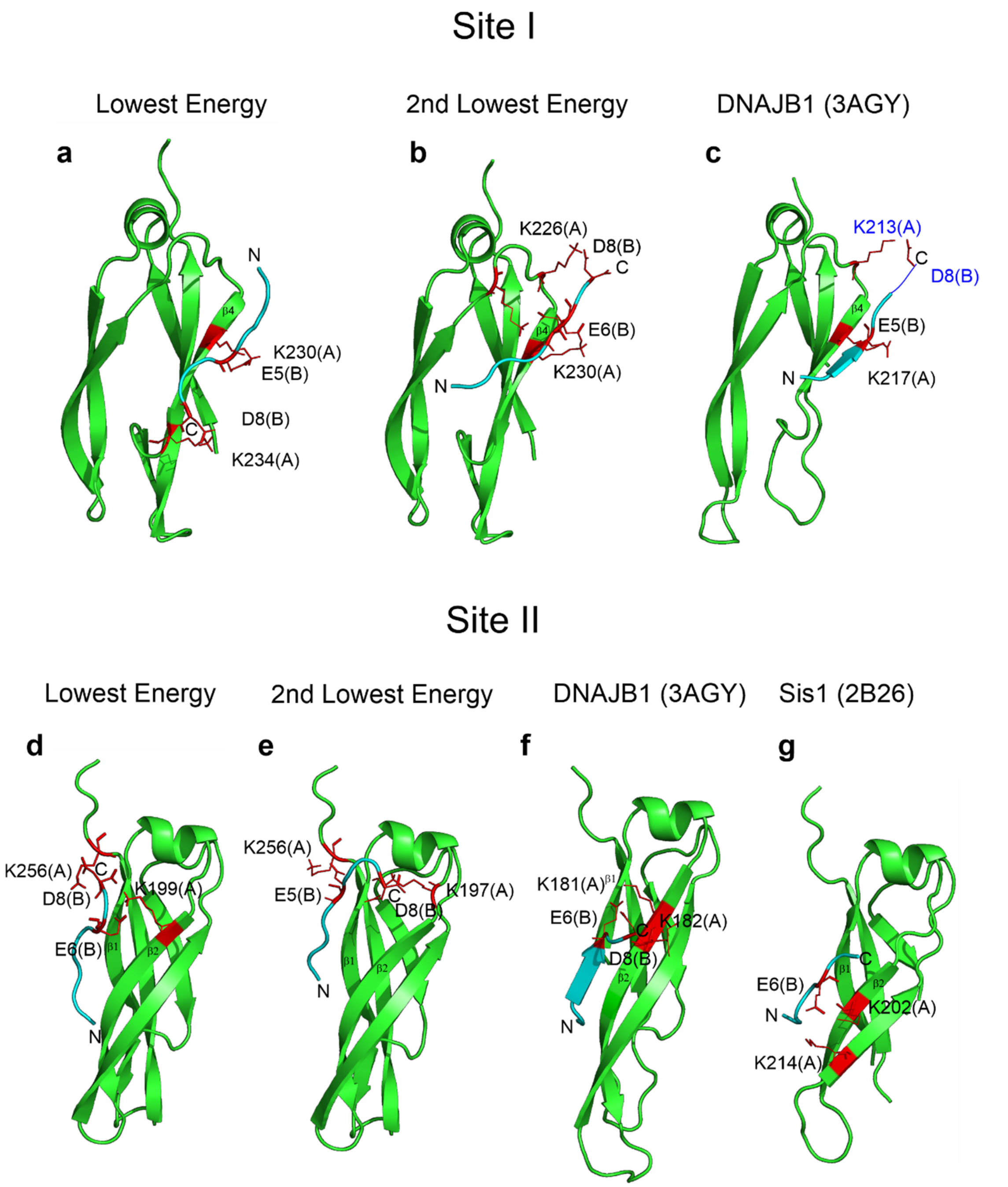

Figure S6. | Position and orientation of the EEVD peptide at CTDI sites I and II of JDPs. For site I, **a** The lowest energy structure from Sis1 (this work) shows that the EEVD peptide has a parallel orientation relative to β4. **b** The second lowest structure diagram from Sis1 (this work) and **c** the crystal structure of DNAJB1 (PDB 3AGY) show that the EEVD peptide has an antiparallel orientation relative to β4. Residues in dark blue are missing in the PDB structure and were drawn. For site II: **d** The lowest and **e** the second lowest structures are shown. The lowest energy structure from Sis1 (in this work) interacts between β1 and β2 of the CTDI. EEVD has the same antiparallel orientation to β2 for **f** in the crystal DNAJB1 (PDB 3AGY) and **g** in the crystal Sis1 (PDB 2B26). The CTDI structures are shown in green, the EEVD peptide is labeled in cyan, and the salt bridges are shown in red.

Site I interaction plot

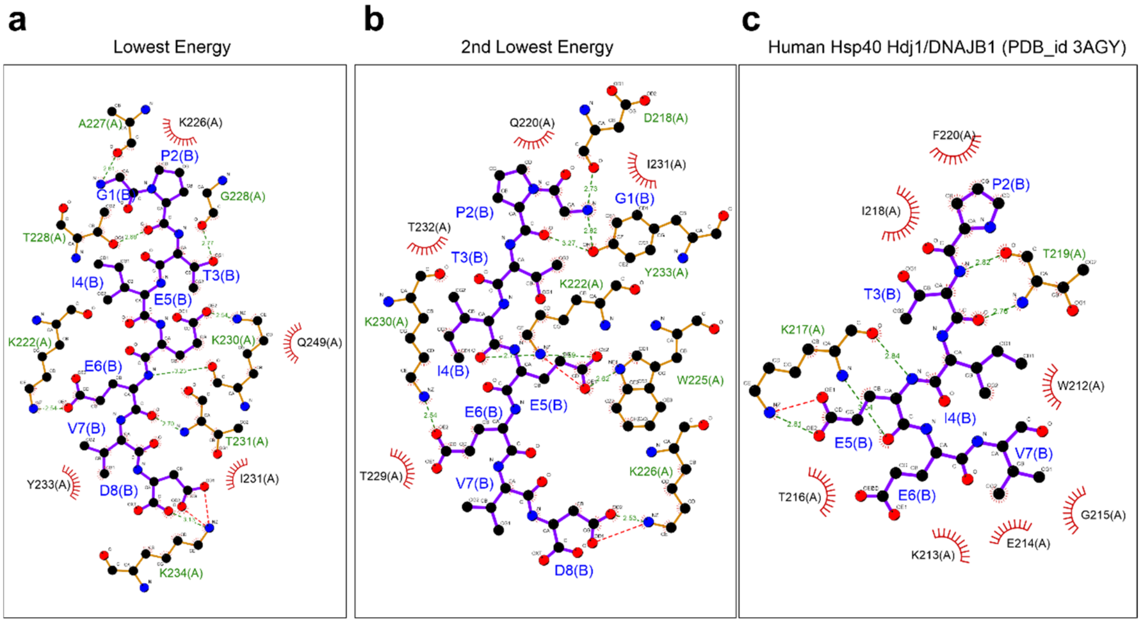

Fig. S7. | EEVD peptide interactions with site I of the JDPs CTDI. a Lowest energy and **b** second lowest energy diagram from Sis1 (this work). **c** Human DNAJB1 (PDB 3AGY). The plots were generated with Ligplot+ v.2.2.4. EEVD peptide residues are shown in blue. Spoked arcs indicate that Sis1 residues are involved in hydrophobic contacts and that green-labeled residues are involved in hydrogen bonds.

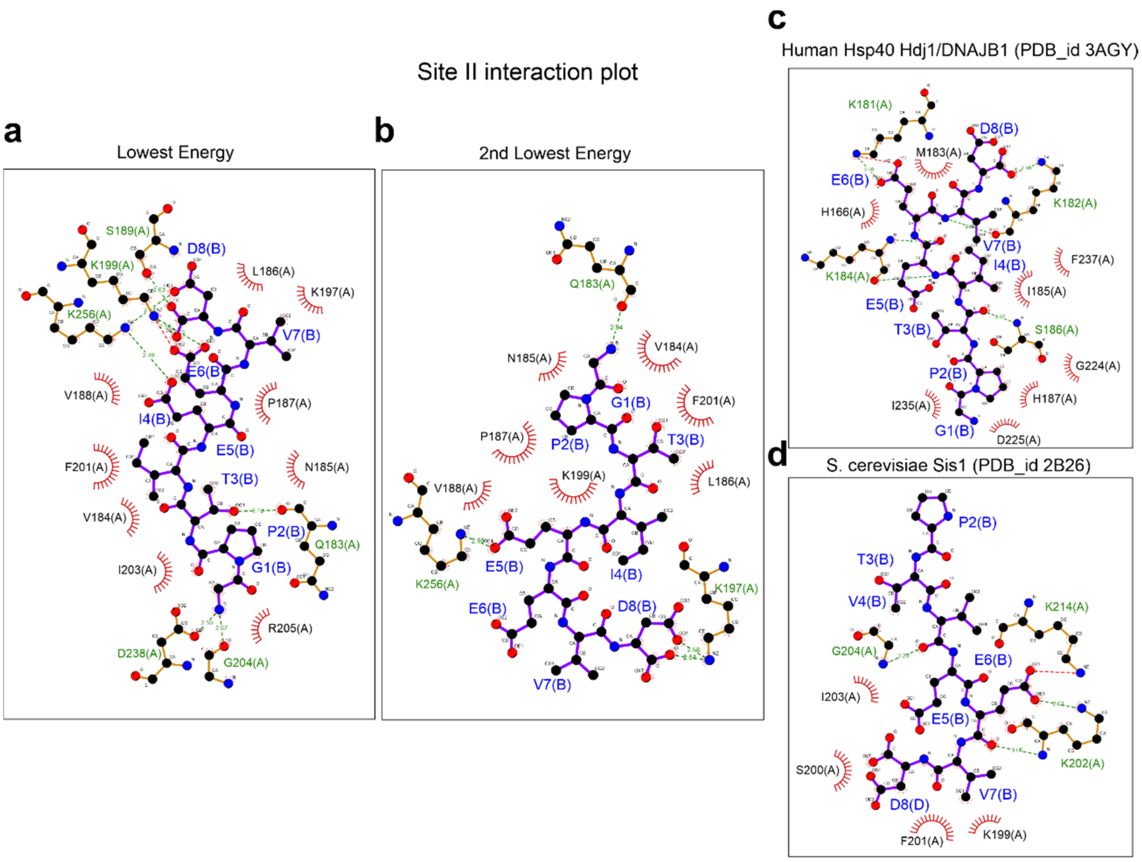

Fig. S8. | EEVD peptide interactions with site II of the JDPs CTDI. a Lowest energy and **b** second lowest energy diagram from Sis1 (this work). **c** Human DNAJB1 (PDB 3AGY). **d** Sis1 (data from PDB 2B26). The plots were generated with Ligplot+ v.2.2.4. EEVD peptide residues are shown in blue. Spoked arcs indicate that Sis1 residues are involved in hydrophobic contacts and that green-labeled residues are involved in hydrogen bonds.

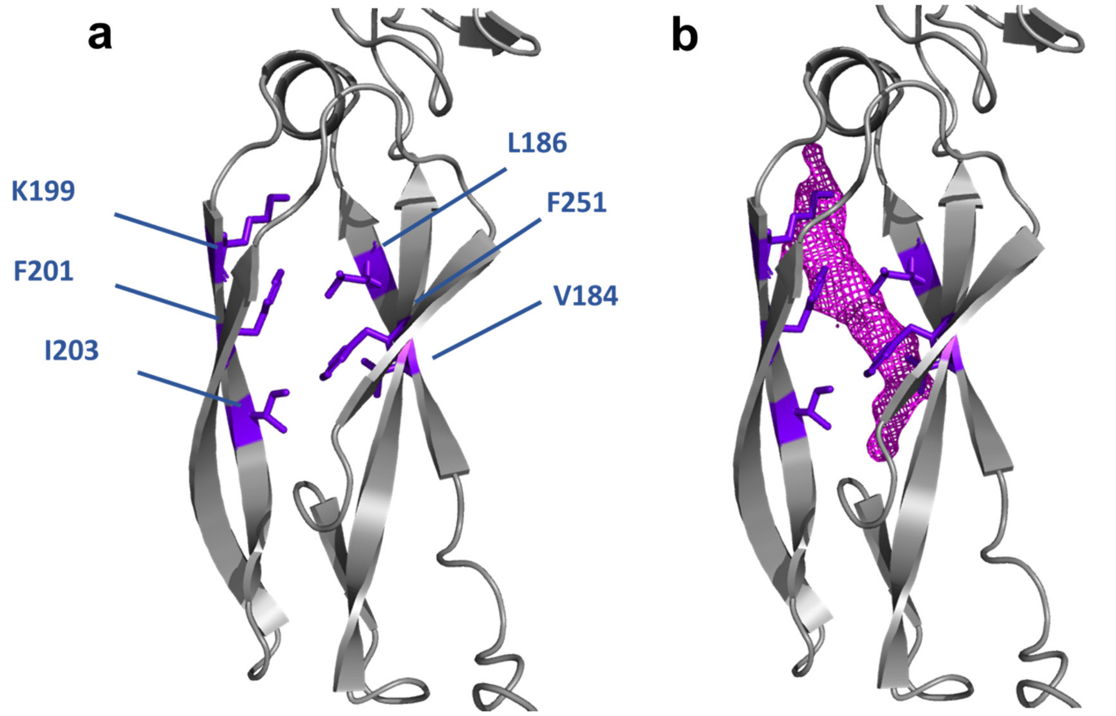

Fig. S9 | CTDI peptide binding site of Sis1. **a** Residues V184, L186, K199, F201, K203, and F251 (blue) were previously described to form a large hydrophobic depression that is likely involved in client protein binding. **b** Atomic probability density map of the EEVD peptide on CTDI-Sis1 (magenta; this work) interacting with the same region described to be involved in binding to the substrate.

A

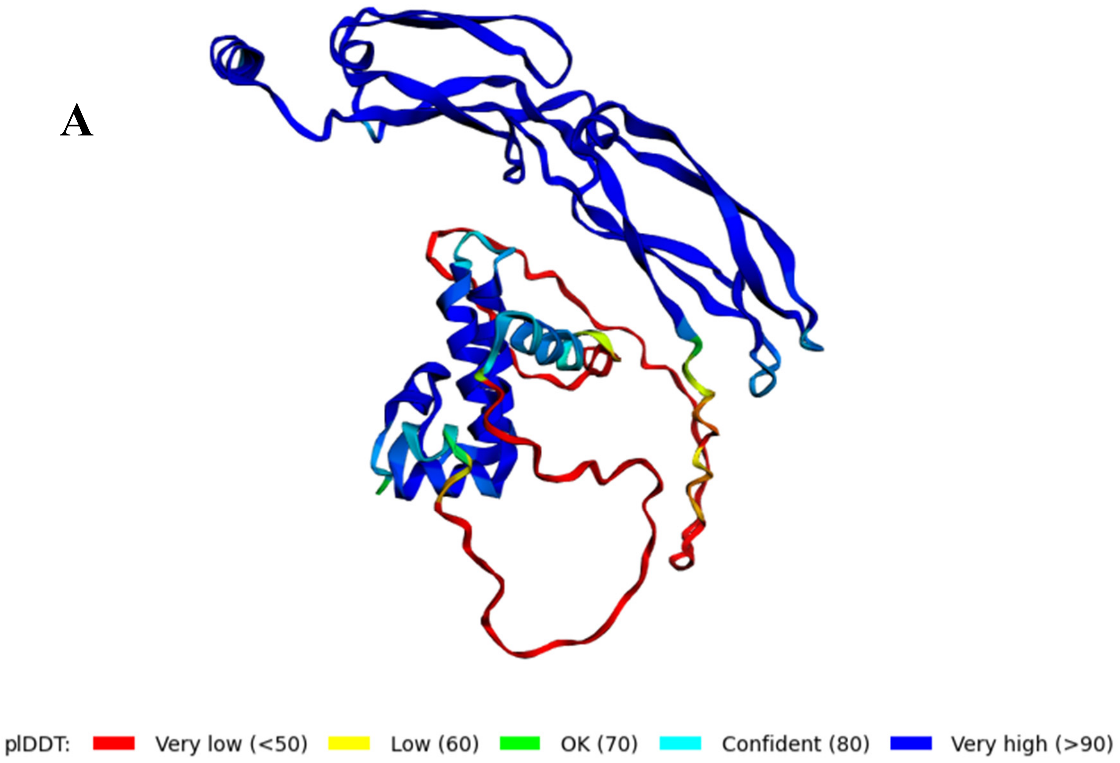
pIDDT:
Very low (<50)
Low (60)
OK (70)
Confident (80)
Very high (>90)

B

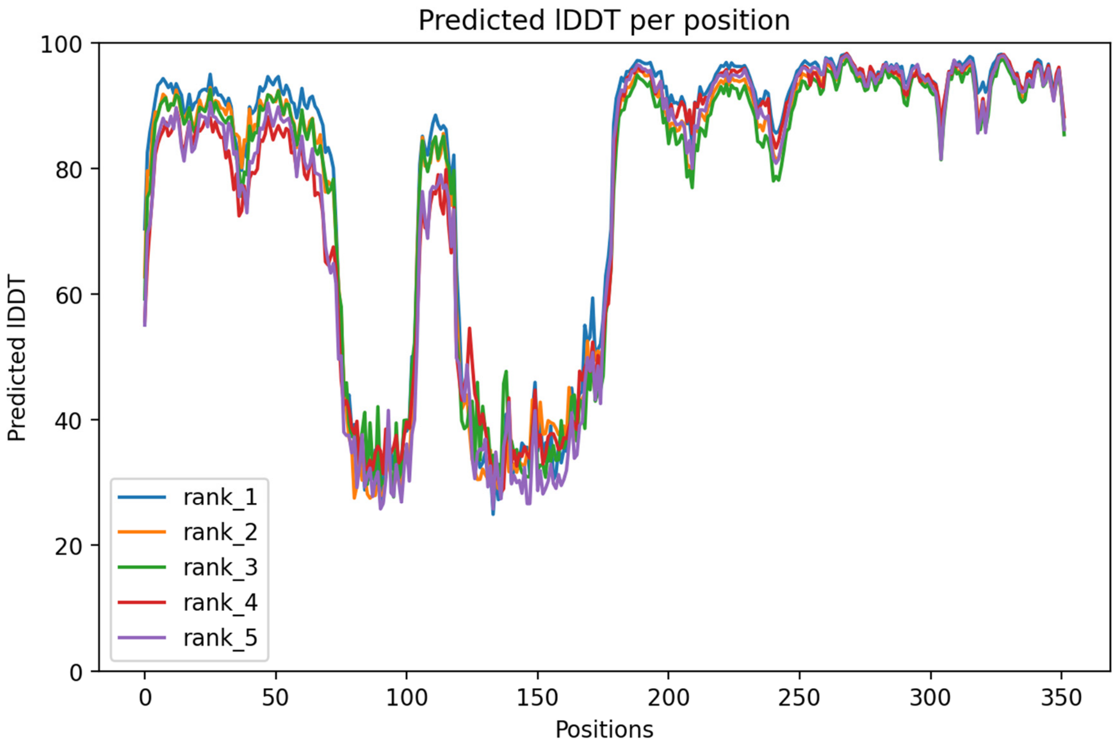
Predicted lDDT per position
Predicted lDDT
Positions
rank_1
rank_2
rank_3
rank_4
rank_5
0
20
40
60
80
100
0
50
100
150
200
250
300
350

C

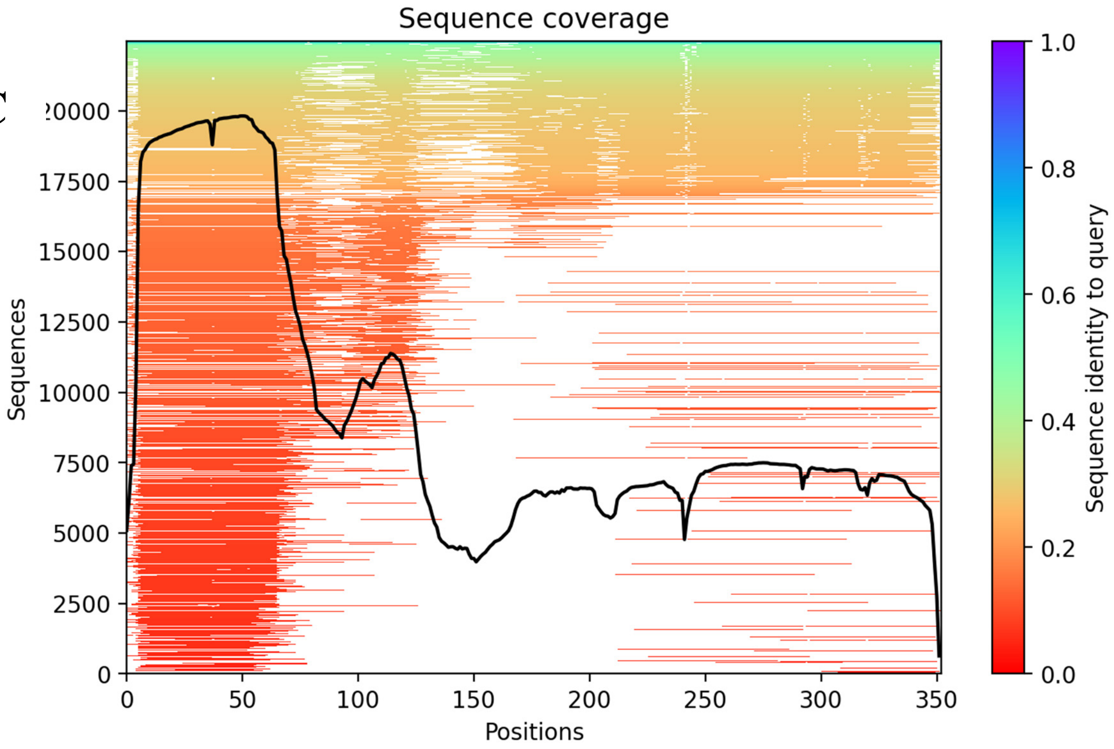

Fig. S10 | Alphafold2 prediction of Sis1. **a** Ribbon representation of the monomer of Sis1 predicted by alphafold2 color coded according to the IDDT (interface predicted Local Distance Difference Test). **b** IDDT per residue of the monomer of Sis1. **c** sequence coverage of the multiple sequence analysis provided by alphafold2.
